# Shared genetic control of expression and methylation in peripheral blood

**DOI:** 10.1186/s12864-016-2498-4

**Published:** 2016-04-06

**Authors:** Konstantin Shakhbazov, Joseph E. Powell, Gibran Hemani, Anjali K. Henders, Nicholas G. Martin, Peter M. Visscher, Grant W. Montgomery, Allan F. McRae

**Affiliations:** Queensland Brain Institute, University of Queensland, Brisbane, QLD Australia; The Institute for Molecular Bioscience, University of Queensland, Brisbane, QLD Australia; QIMR Berghofer Medical Research Institute, Royal Brisbane Hospital, Brisbane, QLD Australia; University of Queensland Diamantina Institute, Translational Research Institute, Brisbane, QLD Australia; Current address: MRC Integrative Epidemiology Unit and School of Social and Community Medicine, University of Bristol, Bristol, BS8 2BN UK

**Keywords:** Gene expression, DNA methylation, Genetic correlation

## Abstract

**Background:**

Expression QTLs and epigenetic marks are often employed to provide an insight into the possible biological mechanisms behind GWAS hits. A substantial proportion of the variation in gene expression and DNA methylation is known to be under genetic control. We address the proportion of genetic control that is shared between these two genomic features.

**Results:**

An exhaustive search for pairwise phenotypic correlations between gene expression and DNA methylation in samples from human blood (*n* = 610) was performed. Of the 5 × 10^9^ possible pairwise tests, 0.36 % passed Bonferroni corrected *p*-value cutoff of 9.9 × 10^-12^. We determined that the correlation structure between probe pairs was largely due to blood cell type specificity of the expression and methylation probes. Upon adjustment of the expression and methylation values for observed blood cellular composition (*n* = 422), the number of probe pairs which survived Bonferroni correction reduced by more than 5400 fold. Of the 614 correlated probe pairs located on the same chromosome, 75 % share at least one methylation and expression QTL at nominal 10^-5^*p*-value cutoff. Those probe pairs are located within 1Mbp window from each other and have a mean of absolute value of genetic correlation equal to 0.69, further demonstrating the high degree of shared genetic control.

**Conclusions:**

Overall, this study demonstrates notable genetic covariance between DNA methylation and gene expression and reaffirms the importance of correcting for cell-counts in studies on non-homogeneous tissues.

**Electronic supplementary material:**

The online version of this article (doi:10.1186/s12864-016-2498-4) contains supplementary material, which is available to authorized users.

## Background

The majority of the significant results from genome wide association studies (GWAS) fall outside of coding regions, leading to the conclusion that the causal variants tagged by many GWAS hits function through the control of genomic regulation, e.g. regulation of gene expression [1]. Not surprisingly, e(xpression) QTLs are frequently employed to prioritize GWAS hits with the aim of linking a variant with a gene; expecting to provide a better biological insight via the wealth of gene based knowledge acquired during the last decades. Likewise, epigenetic marks are employed in a similar manner to get an insight into the possible regulatory mechanism(s) behind GWAS hits. Whereas DNA methylation has been long associated with epigenetic inheritance, recent work clearly demonstrates that genetic factors explain a substantial proportion of the variability of DNA methylation in humans, with the average narrow sense heritability being approximately 0.2 [2]. Similar to eQTLs, m(ethylation)QTLs were mapped in the last five years in a few studies across multiple tissues including both *cis* [2–7] and *trans* [8] SNP to methylation site associations.

The relationship between gene expression and DNA methylation has been long recognized [9, 10]. More recent work has shown both positive and negative correlations between these traits [3–7, 11] in humans. This is consistent with a possible shared genetic control of gene expression and DNA methylation, and indeed the overlap in genetic control of expression and methylation has been assessed in several recent studies [3–5, 11]. There are three types of biological samples examined across these published studies: (i) purified primary cell types employed with a relatively small sample size [5] (ii) tissue (e.g. whole blood) with no control over specific composition of cell types [11], and (iii) immortalized cell lines [3, 4] that often do not represent physiological patterns of expression [12] and methylation in their parental cell type. The aim of this study is to provide an assessment of the shared genetic control between gene expression and DNA methylation while avoiding the above pitfalls.

We employed the Brisbane Systems Genetics Study (BSGS) dataset which has previously been used to estimate the heritability of gene expression and DNA methylation and for e/mQTL mapping [2, 13, 14] in the whole blood. The dataset consists of gene expression, DNA methylation, high-density DNA genotypes and wealth of phenotypes, including whole blood cellular composition. The study is family-based with MZ/DZ twin pairs, their full-siblings and parents. This cohort provides a relatively large sample size, data from primary cells and the ability to control for cell type composition of the whole blood, avoiding many of the pitfalls of previous studies in this area.

## Results

**Fig. 1:**
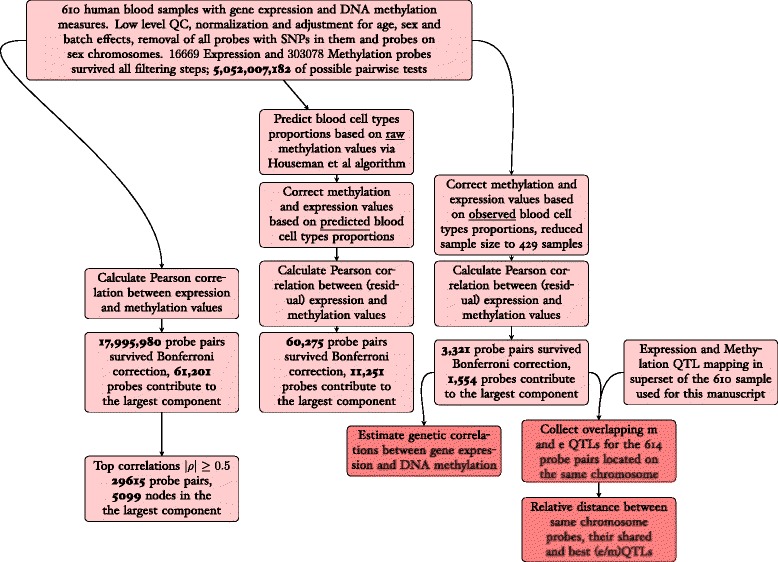
Summary of the analysis workflow

A total of 610 individuals from the BSGS data set had both gene expression and DNA methylation measures as well as high density genotypes [14]. After low-level QC and normalization, expression and methylation probes values were corrected for batch, sex and age effects (see Methods). We removed probes with SNPs in them [2, 15] and probes on sex chromosomes. The final dataset consisted of 16,659 and 303,078 expression and methylation probes respectively that survived all filtering and QC steps, resulting in ≈5 × 10^9^ possible pairwise comparisons (see Fig. 1 for the analysis work flow).

### Cellular composition of the whole blood drives correlation structure between expression and methylation values

The pairwise Pearson correlation (ρ) was calculated between all pairs of expression and methylation probes. Assuming bivariate normal distribution for expression-methylation values for each probe pair, the Fisher Z transformation of the correlation coefficient allows us to obtain asymptotic *p*-values (H0: ρ = 0, H1: ρ ≠ 0) based solely on the correlation coefficient and sample size [16, 17]. The number of probe pairs that survived a Bonferroni correction threshold of 0.05/(16,659 × 303,078) was 17,995,980. In order to simplify initial analysis of the correlation structure, we restricted our attention to probe pairs that pass Bonferroni threshold and have a correlation |ρ|≥0.5. Given a maximum sample size of 610 individuals (ignoring potential missing values for some of the probe pairs) any correlation coefficient |ρ|≥0.27. was deemed significant. The correlation structure among those probes was visualized as a graph where the nodes denote expression/methylation probes and the edges corresponds to a correlation of |ρ|≥0.5 between the probes (Additional file 1: Figure S1). By following the edges from one node to another we can define subsets of connected nodes,known as graph components [18]. The resulting graph consisted of 24 components, with the largest component containing 5099 nodes and 23 small components with median number of nodes equal 2.

We utilized gene expression and DNA methylation data from FACS sorted blood cell types to access the cell-type specificity of expression and methylation probes from the largest graph component. Those purified cell-types data sets consist of DNA methylation data from Reinius et al. [19] and gene expression data from Primary Cell Atlas [20]. The methylation dataset consists of CD19^+^ B cells, CD4^+^ T cells, CD8^+^ T cells, CD56^+^ NK cells, monocytes, eosinophils, and neutrophils cell types and lacks data for basophils in comparison to the cell types that are measured in the BSGS dataset. Multiple lines of evidence were used to show the cell type specificity of the methylation and expression probes from the largest correlation graph component. Firstly, it was observed that 158 out of 500 hematopoietic cell-type specific methylation probes from Houseman et al. [21] are in the largest graph component. We also performed hierarchical clustering of the purified blood cell [19] samples based on the methylation probes from the largest graph component, and this grouped the samples according to their cellular identity (Additional file 2: Figure S2). Similarly, the cell type specificity of the expression probes from the largest correlation graph component was addressed with hierarchical clustering of purified blood cell [20] samples (gene expression levels for B-cells, CD4^+^ T cells, CD8^+^ T cells, NK cells, monocytes and neutrophils were available in the dataset) based on the expression probes from the component and these also grouped samples according to their cellular identity (Additional file 3: Figure S3).

**Fig. 2:**
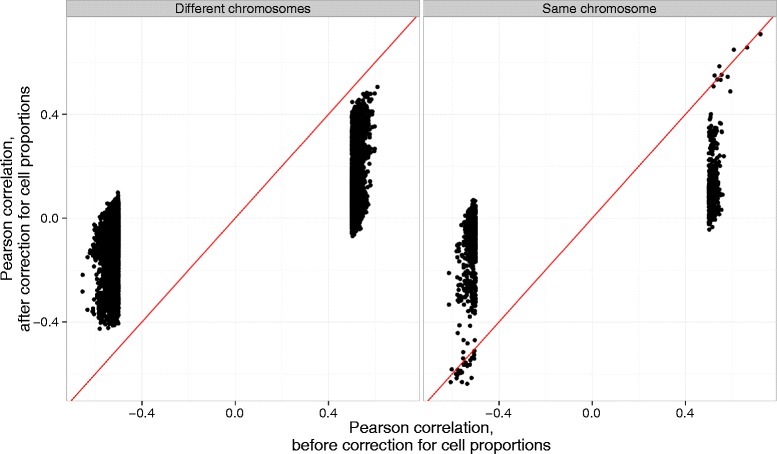
Effect of the adjustment for blood cellular composition on the correlation between expression and methylation. Pearson correlation between gene expression and DNA methylation before (x-axis) and after (y-axis) correction for observed blood cell composition. Probe pairs that passed Bonferroni correction and had a correlation coefficient of |ρ|≥0.5 before adjustment for cellular composition were selected. The probe pairs are split by the relative chromosomal position of the probes in a given pair. The correlations are unchanged after the correction for a small proportion of probe pairs located on the same chromosome. However, the majority of the correlations shifted towards zero. The red line is an identity line

The observation that the largest graph component represented the majority of the probes in the correlation graph, their cell type specificity (i.e. ability to separate purified cell samples into a clusters according to their cellular identity) led to the hypothesis that the differential cell counts among individuals are responsible for the majority of the observed correlation structure. Adjustment of expression and methylation values for nucleated cell proportions was performed in the 422 out of 610 samples in our study that had blood cellular composition measured. The correlations were recalculated based on the adjusted values, which dramatically shifted the mean correlation of the top probe pairs (|ρ|≥0.5 before adjustment) towards zero (Fig. 2).

Looking at a expression-methylation correlations from a previously published study with similar experimental setup [11], we were able to match 2,016 out of their 2,650 and 568 out of their 798 significantly correlated *trans* and *cis* probe pairs respectively in our dataset, where probes were defined as being in *cis* if located within 0.5Mbp of each other on the same chromosome and in *trans* otherwise. The authors did not have access to cell counts, but recognized possibility of cell counts biasing correlation estimates. Indeed, for those probe pairs in our dataset, the majority of their observed correlations shifted towards zero upon adjustment for cellular composition (Additional file 4: Figure S4). The mean of the absolute value of the shift between the correlations before and after the adjustment is 0.23 and 0.34 for *cis* and *trans* probe pairs respectively. Interestingly, a small proportion of the correlations between probes in *cis* (Additional file 4: Figure S4) and same chromosome probe pairs (Fig. 2) were robust to the correction for cell proportions in our data.

### Correcting for cellular composition using predicted cell counts

**Fig. 3:**
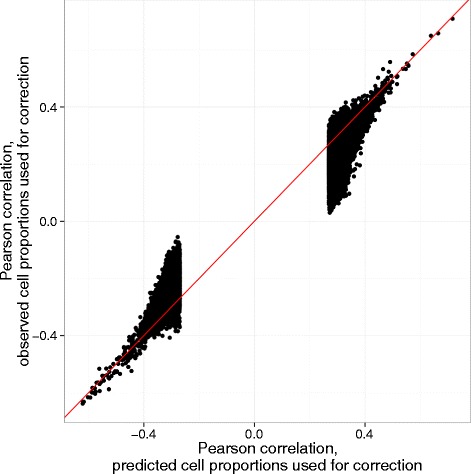
Adjustment for blood cellular composition based on observed vs predicted cell proportions. Pearson correlations between gene expression and DNA methylation levels adjusted for observed (y-axis) and predicted (x-axis) cell proportions. The probe pairs adjusted for predicted cellular composition that passed the Bonferroni significance threshold were selected. The correction for cellular composition was done with either observed or predicted cellular proportions in the 422 and 610 individuals subsets respectively. The red line is an identity line

Whole blood cell counts were only available for the twins and their siblings in the BSGS dataset and not their parents. In order to overcome the reduced sample size due to the availability of blood cellular composition, we predicted cell proportions via previously published algorithm that uses DNA methylation measurements [21] for all 610 samples (see Additional file 5: Figure S5, Additional file 6: Table S1 and Methods). The original method predicts 6 cell types (B cells, CD4 T cells, CD8 T cells, NK cells, monocytes and granulocytes). To obtain finer grained representation of blood composition we re-trained the method on the data from Reinius et al. [19] which allows us to predict 7 cell types (the 8 cell types measured in the 422 subsample minus basophiles, see Methods). Figure 3 shows a comparison of the correlation coefficients corrected for observed (422 sample size) and predicted (610 sample size) cell proportions. Probe pairs for Fig. 3 are selected based on *p*-values (Bonferroni threshold of 9.9 × 10^-12^) from the data corrected on predicted cell proportions.

The shift of the correlations corrected on observed cellular proportions towards zero indicates that using the predicted cellular composition was unable to remove all of the bias in the observed correlations due to differences in cellular composition. In addition we observed large graph component (11,251 probes) in the corrected-on-predicted-proportions graph (Additional file 7: Figure S6), with the majority of probes in that component being once again hematopoietic cell type specific, thus demonstrating an unaccounted for bias due to differential cell counts. This is not surprising given that there will always be some variance that is not accounted for by the predictor. Given the range of correlations between the predicted and observed cellular proportions *r* ≈ 0.75 − 0.95 (Additional file 6: Table S1), we estimated that 10 to 44 % of the original correlation coefficient (no adjustment) remains when using the predicted cellular proportions to adjust expression and methylation values (see Methods). We decided to avoid a high level of false positive calls at the expense of a reduced sample size by restricting our analysis to the 422 individual subset.

### Phenotypically correlated expression and methylation probes

**Fig. 4:**
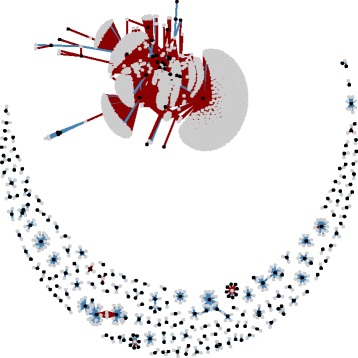
Graph representation of the final correlation structure between gene expression and DNA methylation. Probe pairs with correlation passing a Bonferroni significance threshold after correction for observed cellular composition. Each node represents a gene expression (black) or DNA methylation (grey) probe. Probes connected with red lines are located on difference chromosomes, while blue lines connect probe pairs on the same chromosome. The majority of connections in the largest graph component are between probes on different chromosomes, unlike the rest of the graph mainly represented by connected probes on the same chromosome

In the final 422 individual subset where all the gene expression and DNA methylation measurements are corrected for the observed cell type proportions, there are 3,321 probe pairs that passed the Bonferroni threshold (Additional file 8: Table S4), which map to 232 and 1,922 unique expression (Additional file 9: Table S5) and methylation (Additional file 10: Table S6) probes respectively. Of these probe pairs, 614 are located on the same chromosome and 2,707 otherwise. Again, the graph representation of correlation structure can be split into one largest component (1,554 nodes) and 144 components with median number of nodes equal 4 (Fig. 4). The majority of probes in each probe pair from the largest graph component are located on different chromosomes. This component contains expression probes that related to inflammation and cytotoxic T-cells (e.g. GZMH, CCL5, GPR56) (Additional file 11: Table S2), suggesting another not accounted for confounder such as the inflammation status of an individual.

### Shared QTLs

To avoid potential confounding of unobserved factors such as inflammation, we searched for probes sharing expression and methylation QTLs and thus are likely to be correlated due to common genetic control underlying their variation. We previously estimated heritabilities of expression and methylation probes in the BSGS dataset as well as performed genome wide QTL mapping [2, 14]. Probes in the final list (3,321 probe pairs) are heritable, with the mean heritability of the expression and methylation probes equal to 0.29 and 0.48 respectively (Additional file 12: Figure S7 and Additional file 8: Table S4). For each probe in a probe pair located on the same chromosome, we selected all SNPs with association *p*-value <10^-5^ for both methylation and expression levels. Of 614 same chromosome probe pairs 458 share at least one QTL at this threshold (Additional file 13: Figure S8, Additional file 14: Figure S9, Additional file 15: Figure S10 and Additional file 16: Table S7). These 458 probe pairs map to 135 genomic regions, of which 125 pairs contain expression probe(s) tagging a single gene (Additional file 17: Figure S11). The methylation probes from the probe pairs that do not share QTL(s) tend to be less heritable, with a mean heritability of 0.42 compared to 0.69 for probes with a shared QTL (Additional file 18: Figure S12). Whilst expression probes from both shared and non-shared QTL pairs have mean heritability equal 0.30. A clearer picture is obtained by looking at variance explained by the best m/e SNPs for each probe. The best eSNP(s) (Additional file 19: Table S8) explain 29.4 and 5.4 % of variance on average for the expression probes from probe pairs with and without shared QTL(s) respectively. Likewise, the best mSNP(s) (Additional file 20: Table S9) expain 44.1 and 4.1 % of variance on average for the methylation probes from probe pairs with and without shared QTL(s) respectively (Additional file 21: Figure S13).

The majority of the probe pairs that do not share a QTL contribute to the largest component (144 out of 156 probe pairs), as do probe pairs located on different chromosomes (2,668 out of 2,707 probe pairs). In contrast, same chromosome probe pairs with shared QTL(s) mainly contribute to the small components of the correlation graph (447 out of 458 probe pairs).

**Fig. 5:**
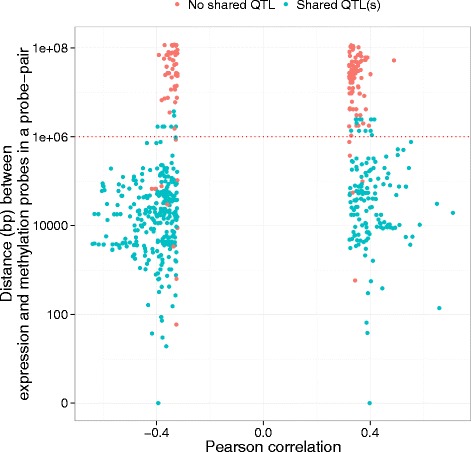
Genomic distance between gene expression and DNA methylation probes for same chromosome probe pairs. Distance between genomic locations of gene expression and DNA methylation probes in a same chromosome probe pair and the phenotypic correlation between them split by shared QTL(s) status. Same chromosome probe pairs (614 probe pairs) from the final correlation list were selected. Red and green points represent probe pairs without and with shared QTL(s) respectively. The red dashed line is at 1Mbp

Of the probe pairs that share QTL(s), 95.5 % are located within 1Mbp of each other, whilst only 11.5 % of pairs that do not share a QTL located within this distance. Probe pairs with and without shared QTL(s) have a mean absolute value Pearson corelation of 0.41 and 0.34 respectively (Fig. 5).

**Fig. 6:**
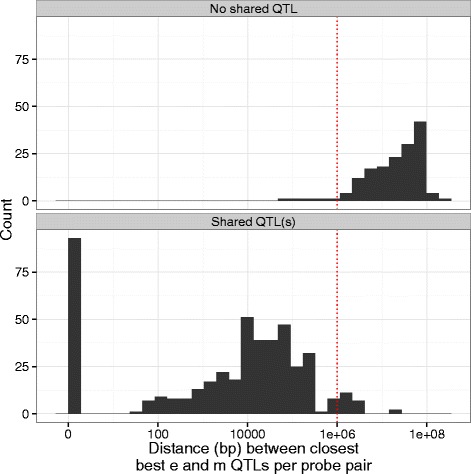
Distance between the nearest best methylation and expression SNPs per same chromosome probe pair. Distribution of the distance between the nearest most significantly associated methylation and expression SNPs for same chromosome probe pair split by shared QTL(s) status. No requirement of significance level was enforced on the most associated SNP. Of 458 probe pairs that share QTL(s) 93 share their best SNPs, i.e. it is the same SNP which is both best expression and best methylation SNP for probes in a probe pair. Red dashed line is at 1Mbp

We observed great variability in the number and proportion of shared SNP(s) associated with the expression and methylation levels of a pair which likely represents LD structure at each genomic location (Additional file 13: Figures S8 and Additional file 22: Figure S14). There was 93 probe pairs (47 genomic regions) that had the same best SNP from m and e QTL association mappings. The majority of the probe pairs with shared SNP(s) (95.6 %) have their best m and e SNPs within 1 Mbp window, unlike the probes without a shared QTL (3.2 %, Fig. 6).

### Concordance in genetic control of expression and methylation levels

**Fig. 7:**
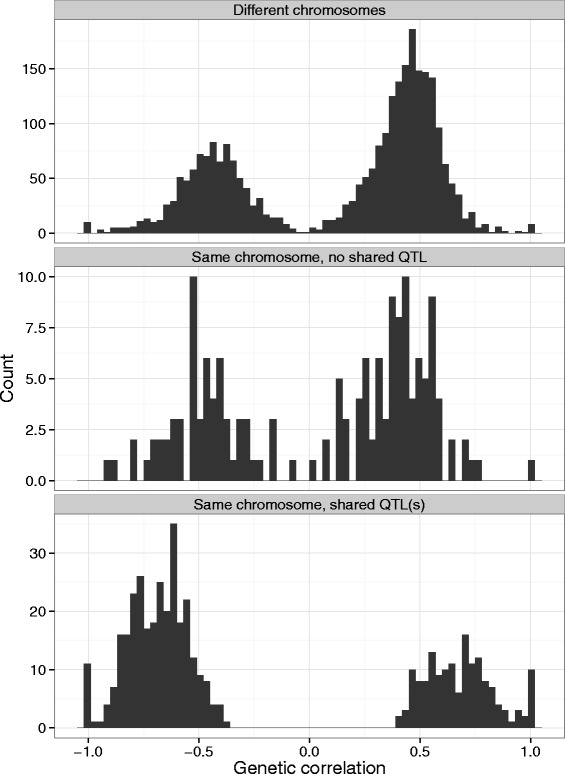
Distribution of genetic correlation estimates between gene expression and DNA methylation. The estimates split by relative chromosome location and shared QTL status. Probe pairs from the final correlation list (3321 probe pairs) were selected. The distribution of the correlations is similar for probe pairs located on different chromosomes and same chromosome probe pairs without shared QTL. Probe pairs that share QTL(s) have larger genetic correlations than the rest of probe pairs

We estimated genetic correlations between DNA methylation and gene expression for all probe pairs passing Bonferroni correction threshold (3,321 pairs) with bivariate gREML utilizing SNP based genomic relationship matrix [22, 23] (GRM) (Fig. 7). It is important to note that in this settings SNP based GRM reconstitutes pedigree structure of the BSGS dataset. Probe pairs that share QTL(s) have greater mean genetic correlation −0.69/0.68 (for positive and negative peaks respectively) in contrast to −0.48/0.4 and −0.45/0.45 for same chromosome probe pairs with no shared QTL and probe pairs on different chromosomes respectively (Fig. 7 and Additional file 8: Table S4).

## Discussion and conclusions

We have quantified the overlap in the regulation of gene expression and DNA methylation by looking at genome-wide phenotypic correlations and their underlying cause and by estimating genetic correlation in a mixed model setting.

Unlike SNP genotypes, both gene expression and DNA methylation are affected by environmental factors and by the cell type in which they are measured. It has been recognized that the cellular composition of a tissue under investigation can bias epigenetic association studies [24]. Blood is a multicellular tissue and there is a continuum of scenarios from cell-type specific to cell-type uniform levels of gene expression and DNA methylation. Even though there is only a small number (*N*_*meth*_ and *N*_*expr*_) of DNA methylation and gene expression probes affected by differential cell counts [2, 14] there are *N*_*meth*_ × *N*_*expr*_ pairs of those probes. The small but consistent association of a particular probe value with a cell type proportion can often be regarded as negligible in a GWAS study, however association between two probes of that kind will produce a substantial correlation coefficient. It is therefore not surprising that cell type specific gene expression and DNA methylation are indeed at the top of the correlation structure we observed in blood.

Taking advantage of a cell type proportion predictor [21] allows us rectify the problem to a great extent with an almost 300 times reduction in number of probe pairs surviving Bonferroni correction on their estimated correlations. However as with any imperfect predictor there is unaccounted for variance that still causes a problem when we look at the covariance between the traits. Given we cannot expect each and every experiment on a complex tissue to have cellular composition recorded it is instructive to understand how much of a correction we can achieve with a given predictor and what other filtering steps might help in such situations. To that extent, we estimated the amount of unaccounted for covariance and utilized genetic data to further filter our probe pairs. Moreover additional methylation data on finer grained cell types (e.g. basophils) will further help in predicting blood cellular compositions, which in turn should improve our ability to control for the cellular heterogeneity in blood.

A graph representation of the correlation structure proves to be helpful in depicting relationships between the probes and confounding. The number of nodes in the largest graph component allowed us to judge the amount of confounding, as it is expected that majority of the correlations should be independent from each other given the power we can achieve with our sample size. The majority of correlated probes joined together to form a large graph component representing 99.6 % of all nodes in the original graph, which decreases to 91.6 % and 72.1 % when adjusted for predicted and observed cell counts respectively. At first we thought that removing the largest component and working with the small order components would preserve the true positives and remove confounded signal. However, the majority of the same chromosome probe pairs with shared QTL(s), and thus genuine correlations, were found to be located within the largest component when the traits were not corrected for cell counts (Additional file 23: Table S3). The expression and methylation probes from the largest component provided strong evidence that the nature of confounding in correlation measures was primarily due to cell count differences. Furthermore the largest component in the graph of cell type corrected correlations consists of probes that are located on different chromosomes or same chromosome probes without shared QTL. Gene expression probes in the largest component tag inflammation related genes, indicating either the presence of confounding via an inflammation status of an individual or a need for the adjustment for cell proportions with a non-linear model. However, whilst it is unlikely, we cannot exclude the presence of a genuine master regulator (e.g. transcriptional factor that regulates expression of all the genes in the component) driving this correlation structure.

Gene expression and DNA methylation are heritable and for at least some loci these have large QTL explaining the majority of their phenotypic variation. Hence some SNPs with large effect sizes are found in association studies, with the majority of the associated SNPs located within the *cis* region. We hypothesized that if the phenotypic correlation is mainly driven by a genetic component, then we expect to find SNPs that explain a substantial proportion of variance for both traits. At the same time, given genotypes are not affected by environmental factors or cellular composition, we expect the phenotypic correlation to be reflective of the genotypic correlation and therefore not confounded (or less confounded).

The probes that are located on the same chromosome but do not share a QTL tend to have their best m and e SNPs further apart (>1Mbp) and on average explain 5 (expression) and 10 (methylation) times less variance compared to the best SNPs for probes with shared QTL(s). Altogether, this indicates that the most associated SNPs for probes without shared QTL are background noise and not genuinely associated QTL. Interestingly, the bulk of same chromosome probes that share at least one QTL fall within 1Mbp window that is often used as the definition of a *cis* relationship between genetic ranges.

Finally, for each probe pair that passed the Bonferroni threshold, we estimated the genetic covariance between DNA methylation and gene expression in mixed model settings. Unlike single SNP QTL analysis, bivariate gREML captures covariance attributable to the all imputed SNPs (which is reflective of the pedigree structure in our settings). In the case of single SNP analysis we are unable to discover all of the e/mQTLs, due to lack of statistical power, whilst gREML provides us with overall (genome-wide) estimate of total genetic covariance between DNA methylation and gene expression. As expected, probe pairs with shared QTL(s) on average have larger genetic correlations in contrast with the same chromosome probe pairs without shared QTL. We attribute the non-zero genetic correlations for the same chromosome pairs without shared QTL and probe pairs on different chromosomes to be due to selection bias. That is to say, the probes have phenotypic correlation greater than a certain threshold because we selected them to pass the Bonferroni threshold.

We have clearly demonstrated the effect of the cell heterogeneity on the correlations between DNA methylation and gene expression levels and shown that correction for predicted cell counts is not sufficent to remove these effects. Another example where cell heterogeneity possibly plays a role is a case–control studies, where it is possible to find different cell composition of blood (and/or other tissues) due to the disease status of affected patients (e.g. due to inflamation). This situation can be further complicated in that predictions developed using normal (control) cells, may not fully capture the range of cellular content in case samples. In such situation, using observed cell proportions is the best way to perform analysis.

Overall we have showed the importance of the need to control tissue heterogeneity for studies of gene expression and DNA methylation and employed a graph representation of the correlation structure to gauge possible bias. This and shared e/mSNP(s) provided us with a sound basis to select probe pairs to demonstrate shared genetic control between DNA methylation and gene expression.

## Methods

The overall work flow of the analysis is presented in Fig. 1.

### Ethics statement

Written, informed consent was obtained from all participants, including a parent or guardian for those aged under 18 years, and the study was approved by the Human Research Ethics Committee at the QIMR Berghofer Medical Research Institute.

### Brisbane systems genetics study

The BSGS dataset consists of microarray measurements of whole blood gene expression and DNA methylation, high-density SNP genotypes and wealth of phenotypes including whole blood cellular composition. The BSGS study is family-based with MZ/DZ twin pairs, their full-siblings and parents [2, 12–14]. We employed a subset of 610 individuals from 117 families that have both gene expression and DNA methylation measured as well as SNP genotypes.

DNA samples were genotyped on the Illumina 610-Quad Beadchip. SNPs were called with Illumina BeadStudio software. After standard QC, we removed SNPs with minor allele frequencies (MAF) < 1 % and mean BeadStudio GenCall < 0.7 % leaving 528509 SNPs. These SNPs were phased with HAPI-UR [25] and imputed against 1000 Genomes [26] (V1.3; hg19) data with Impute V2 [27, 28]. Imputed SNPs were filtered to discard SNPs with *r*^2^ <0.8 , MAF < 0.05, missing rate > 10 % and HWE *p*-value < 10^-6^, resulting in 6005138 imputed SNPs. For a full description of genotyping and inputation see Medland et al. [29] and Powell et al. [13].

Whole blood gene expression levels were measured with the Illumina HT12-v4.0 bead array as described in Powell et al. [14]. Briefly, gene expression was background corrected, log2 transformed and quantile normalized. Batch, sex and age effects were removed by taking residuals from a linear model.

Whole blood DNA methylation levels were obtained with the Illumina HumanMethylation450 bead array and normalized as described in McRae et al. [2]. Briefly, no global normalization was performed as (e.g.) quantie normalisation may remove genetic and environmental effects that act globally on methylation. Methylation β-values were transformed to M values when adjusted for batch, sex and age effects by taking residuals from a generalized linear model with logistic link function.

Estimation of heritability and QTL mapping was performed on the larger set of 614 and 862 individuals from the BSGS dataset for DNA methylation and gene expression respectively. Gene expression heritability was estimated by partitioning phenotypic variance (*V*_*P*_) into additive genetic (*V*_*A*_), common family (*V*_*f*_) and environmental (*V*_*E*_) components. DNA methylation heritability was estimated by partitioning phenotypic variance (*V*_*P*_) into additive genetic (*V*_*A*_) and environmental (*V*_*E*_) components. Variance component models were fitted with QTDT [30]. Association analysis between imputed SNPs and gene expression or DNA methylation was performed with FASTASSOC component of MERLIN [31]. See Powell et al. [13, 14] and McRae et al. [2] for a full description.

### Pearson correlations and asymptotic *p*-values

For the correlation analysis we disregarded gene expression and DNA methylation probes on the sex chromosomes. After all QC and filtering steps, there are 16,659 and 303,078 gene expression and DNA methylation probes remaining respectively. Pearson correlations between gene expression and DNA methylation values were calculated utilizing all individuals with non-missing pairwise measurements for each probe-pair. Assuming bivariate normal distribution for expression-methylation values in a probe pair, the Fisher Z transformation of correlation coefficient allows us to obtain asymptotic *p*-values (H0: ρ = 0, H1: ρ ≠ 0) based solely on correlation coefficient and sample size [16, 17].

An R package was developed to handle the large size of the correlation matrix, which uses memory mapped files as a storage back-end through the R [32] ff library [33] and parallel block-wise operations on matrices to reduce processing time. This is available in the ffbw R package (https://github.com/kn3in/ffbw).

### Cell type specificity of expression and methylation probes

DNA methylation and gene expression data of FACS purified hematopoietic cells was obtained from Reinius et al. [19] and the Primary Cell Atlas [20]. The methylation data was accessed through Bioconductor FlowSorted.Blood.450 k package. The dataset consists of CD19^+^ B cells, CD4^+^ T cells, CD8^+^ T cells, CD56^+^ NK cells, monocytes, eosinophils, and neutrophils cell types. Gene expression data was accessed through www.biogps.org REST API. The original data was obtained on Affymetrix platform therefore we mapped Illimuna expression probes ids to Affymetrix expression probe ids via Bioconductor biomaRt package. Gene expression levels for B cells, CD4^+^ T cells, CD8^+^ T cells, NK cells, monocytes and neutrophils were available in the dataset. Hierarchical clustering of samples of purified cells was performed using the expression or methylation levels of the probes from the largest correlation graph component. The clustering dendrogram was plotted alongside of a heatmap of expression or methylation levels in the purified cells.

For each probe on the methylation array and each cell type in the Reinius [19] dataset we obtained rank based on differential methylation of a probe between a given cell type and the rest of the cell types. The differential methylation was called on β-values with Bioconductor limma package.

### Correction for cellular proportions

Measurements of nucleated blood cell counts per given volume of blood for CD19^+^ B-cells, CD4^+^ T cells, CD8^+^ T cells, CD56^+^ NK cells, monocytes, eosinophils, basophils and neutrophils were available for 422 individuals in the BSGS dataset. The observed proportions for the 8 cell types per individual sample in the BSGS dataset were calculated as a ratio of cell counts for a given cell type divided by the total count of all 8 cell types in that individual sample.

Gene expression and DNA methylation levels were corrected for variation in blood cell type proportions by taking residuals from the linear model *y* = *X*β + *e* where β vector represent effects of each cell type, *y* is the normalized methylation or expression value and *X* is a design matrix. The proportions used in the adjustment were either predicted via Houseman et al. method [21] (sample size 610) or observed proportions (sample size 422).

The cell proportions were predicted with the Houseman et al. algorithm [21] re-trained on Reinius dataset [19] as follows:

(i) Selected probes with the cell type specificity rank (differentially methylated probes described above) less or equal to 70 for each cell type, giving 560 probes in total of which 529 present in the BSGS methylation dataset after QC (Additional file 24: Figure S15).
(ii) For each probe, the estimated mean methylation level per cell type was used in the predictor.
(iii) Raw uncorrected methylation β values of the selected probes were used to predict cellular proportions without requiring either individual predicted cell proportions be greater than zero or sum of all cell proportions per sample equal to one. The former is due to the fact that restricting predicted proportions to be non-negative shifted proportion of Eosinophils to zero for many samples and the latter is because the 7 cell types do not represent all nucleated blood cells (nor can any other number of cell types can be claimed as precise definition of blood composition, however the 8 cell types represent majority of the nucleated cells in the blood and hence one can require their proportions to sum to one). Performance of the predictor was measured by the Pearson correlation between the observed and predicted proportions (Additional file 6: Table S1) in the 422 samples subset.
(iv) Predicted proportions were calibrated on observed values (422 samples) such that regression of predicted values on observed values has a slope equal one and an intercept equal zero (Additional file 5: Figure S5), which puts the predicted proportions on a meaningful scale.

### Estimation of genetic correlations and concordance of m and e QTL signals

Genetic correlations between DNA methylation and gene expression were calculated via bivariate gREML as implemented in the GCTA [22, 23] software package. The genomic relationship matrix (GRM) for all individuals in the dataset (610 samples) was calculated using imputed genotypes.

For each probe from the same chromosome probe pair list (614 probe pairs), we collected all m and e QTL mapping calls located on the same chromosome as the probe pair. We then queried all associated SNPs at a nominal 10^-1^*p*-value cut-off to collect best SNP (lowest *p*-value) per probe (relaxed cutoff given some of the probes do not have m/eQTLs in a sense of passing a genome wide significance threshold). Next we restricted all SNPs to have a *p*-value <10^-5^ and selected the overlapping m and e SNPs per probe pair. These SNPs were used to count number of shared associated SNP(s) per probe pair (Additional file 25).

### Probes and QTLs annotation

Expression and methylation probes positions on the hg19 genome assembly were obtained through Bioconductor libraries illuminaHumanv4.db and FDb.InfiniumMethylation.hg19 respectively. SNPs positions were mapped to the hg19 genome assembly through their reference id numbers via ensembl REST API. All genomic ranges manipulations were performed via Bioconductor GenomicRanges library.

## Availability of supporting data

The gene expression and DNA methylation data are available at the Gene Expression Omnibus under GSE53195 (http://www.ncbi.nlm.nih.gov/geo/query/acc.cgi?acc=GSE53195) and GSE56105 (http://www.ncbi.nlm.nih.gov/geo/query/acc.cgi?acc=GSE56105) IDs respectively.

## Additional files

Additional file 1: Figure S1. Graph representation of the top correlation structure. Black and gray nodes represent gene expression and DNA methylation probes respectively. Edges represent correlations ≥0.5 that survived Bonferroni correction. The Pearson correlation between gene expression and DNA methylation was calculated based on levels unadjusted for cellular composition in 610 individuals from the BSGS dataset. The majority of the probes are connected to form the largest graph component. (PDF 2191 kb) Additional file 2: Figure S2. Heatmap of DNA methylation (rows) matrix across purified hematopoietic cell types (columns). The methylation probes are selected based on the largest component of the top correlation graph (|ρ|≥0.5) before adjustment for the blood cellular composition. Hierarchical clustering separates samples into clusters according to their cell identity. The methylation data from Reinius et al. [19]. (PDF 1219 kb) Additional file 3: Figure S3. Heatmap of gene expression (rows) matrix across purified hematopoietic cell types (columns). The genes are selected based on the largest component of the top correlation graph (|ρ|≥0.5) before adjustment for blood cellular composition. Hierarchical clustering separates samples into clusters according to their cell identity. Cell type identity of samples encoded by color bar at the top of the heatmap. The data from primary cell atlas [20]. (PDF 583 kb) Additional file 4: Figure S4. Correlation look up of previously published expression-methylation probe pairs. Pearson correlations between gene expression and DNA methylation before (610 individuals) and after (422 individuals) adjustment for cellular composition in the BSGS dataset for *cis* and *trans* probe pairs that showed significant association in Eijk et al. [11]. The majority of the correlations shifted toward zero upon the adjustment for cellular composition. (PDF 6 kb) Additional file 5: Figure S5. Relationship between the observed (x-axis) and predicted (y-axis) cell proportions in the BSGS dataset split by cell type. Cell proportions were predicted utilizing methylation data with Houseman et al. [21] method re-trained on Reinius et al. [19] dataset. The predicted proportions were calibrated on the 422 subsample that have cellular composition measured to have a slope equal one and an intercept equal zero when regressed on observed proportions (see Table S1 for correlations between observed and predicted proportions). The red line is an identity line. (PDF 27 kb) Additional file 6: Table S1. Pearson correlations between observed and predicted cell proportions in the 422 individuals subset of the BSGS dataset. The predicted cell proportions are estimated via Houseman et al. method [21] which was retrained on Reinius et al. data [19]. (DOC 27 kb) Additional file 7: Figure S6. Distribution of the number of nodes per graph component. Expression and methylation levels were adjusted for predicted cell proportions. The correlation graph was constructed from probe pairs (60275 probe pairs) passing Bonferroni correction. The largest component consists of 11251 nodes. (PDF 4 kb) Additional file 8: Table S4. The 3321 probe pairs from the final correlation list. Phenotypic Person correlation and genetic correlations between expression and methylation probes, related statistics and heritabilities are provided. (CSV 390 kb) Additional file 9: Table S5. Annotation and genomic position of the expression probes from the final correlation list. (CSV 26 kb) Additional file 10: Table S6. Genomic position of the methylation probes from the final correlation list. (CSV 97 kb) Additional file 11: Table S2. Number of unique DNA methylation probes correlated with a gene in the largest correlation graph component. Probe pairs from the largest graph component of the final correlation list were selected (see Fig. 4). Unique methylation probes per gene were counted such that a methylation probe was counted only once if it correlated with more than one expression probe tagging the same gene. (DOC 33 kb) Additional file 12: Figure S7. Distribution of the heritability of expression and methylation probes from the final list of correlated probe pairs (3321 probe pairs). Unique expression and methylation probes were extracted from 2707 different chromosome and 614 same chromosome probe pairs. (PDF 6 kb) Additional file 13: Figure S8. Distribution of number of overlapping QTLs per probe pair. For each of the 614 same chromosome probe pairs, expression and methylation QTLs at nominal 10^-5^ *p*-value threshold were selected. The number of overlapping SNP was counted for each probe pair. 156 pairs do not share QTL. (PDF 4 kb) Additional file 14: Figure S9. Scatterplot of gene expression and DNA methylation for top sixteen same chromosome probe pairs with shared QTL(s). Red line is a linear regression of DNA methylation on gene expression values. (PDF 65 kb) Additional file 15: Figure S10. Scatterplot of gene expression and DNA methylation for same chromosome probe pairs with shared QTL(s). Color represents number of alleles of e/mSNP shared by a DNA methylation gene expression probe pair. (PDF 16 kb) Additional file 16: Table S7. Results of the association analysis between shared e/mSNPs and same chromosome probe pairs with a shared QTL(s). After selecting all the overlapping SNPs at nominal 10^-5^ association *p*-value threshold, we pruned the list to contain only SNPs with the lowest *p*-value from both the expression and methylation association analysis. (CSV 130 kb) Additional file 17: Figure S11. Graph representation of the correlation structure for the same chromosome probe pairs with shared QTL(s). The graph is constructed by utilizing selected probes from the final correlation list. There are 135 components each of which correspond to a unique genomic location tagged by probes. 125 components correspond to a single gene. Expression and methylation probes are represented by black and grey nodes respectively. Positive and negative correlations are depicted as red and black edges respectively. (PDF 38 kb) Additional file 18: Figure S12. Distribution of heritability of expression and methylation probes from same chromosome probe pairs (614 probe pairs, the final correlation list) split by shared QTL(s) status. Unique methylation and expression probes were extracted from 458 same chromosome shared QTL(s) and 156 same chromosome no shared QTL probe pairs. (PDF 5 kb) Additional file 19: Table S8. Best association eSNPs for the expression probes from the final correlation list. (CSV 37 kb) Additional file 20: Table S9. Best association mSNPs for the methylation probes from the final correlation list. (CSV 121 kb) Additional file 21: Figure S13. Distribution of probe variance explained by the best SNP. For each unique expression and methylation probe from the same chromosome probe pair list (614 probe pairs) best expression and methylation association SNP respectively were selected. Probes were split based on the shared QTL status of the probe pair they originate from. (PDF 6 kb) Additional file 22: Figure S14. Distribution of proportion of shared QTLs per probe pair. The 458 same chromosome probe pairs with shared QTL(s) from the final correlation list were selected. The proportion was calculated as the ratio of number expression and methylation association SNPs with the same rs id number at 10^-5^ *p*-value cutoff (shared QTL(s)) to the number of all unique m and e SNPs at the same threshold per probe pair. (PDF 4 kb) Additional file 23: Table S3. The number of the same chromosome probes pairs with shared QTL(s) from the final correlation list that contribute towards different graph components stratified by the adjustment methods. The last row of the table is the final correlation graph (Fig. 4). (DOC 27 kb) Additional file 24: Figure S15. Heatmap of DNA methylation (rows) matrix across purified hematopoietic cell types (columns). The methylation probes are selected based on differential methylation calls between a given cell type and the rest of the cell types. Probes with rank smaller or equal 70 selected. The methylation data from Reinius et al. [19]. (PDF 895 kb) Additional file 25: Modeling of correlation induced by differential cell counts and column description of the Tables S4-S9. (DOCX 90 kb)

## Abbreviations

BSGS: Brisbane systems genetics study
eQTL: Expression QTL
GRM: Genomic relationship matrix
GWAS: Genome wide association studies
MAF: minor allele frequencies
mQTL: Methylation QTL
