# Supplementary figures and images for: Shared genetic control of expression and methylation in peripheral blood
Source: BMC Genomics. 2016 Apr 6;17:278. doi: 10.1186/s12864-016-2498-4 (PMC4822256; doi:10.1186/s12864-016-2498-4)

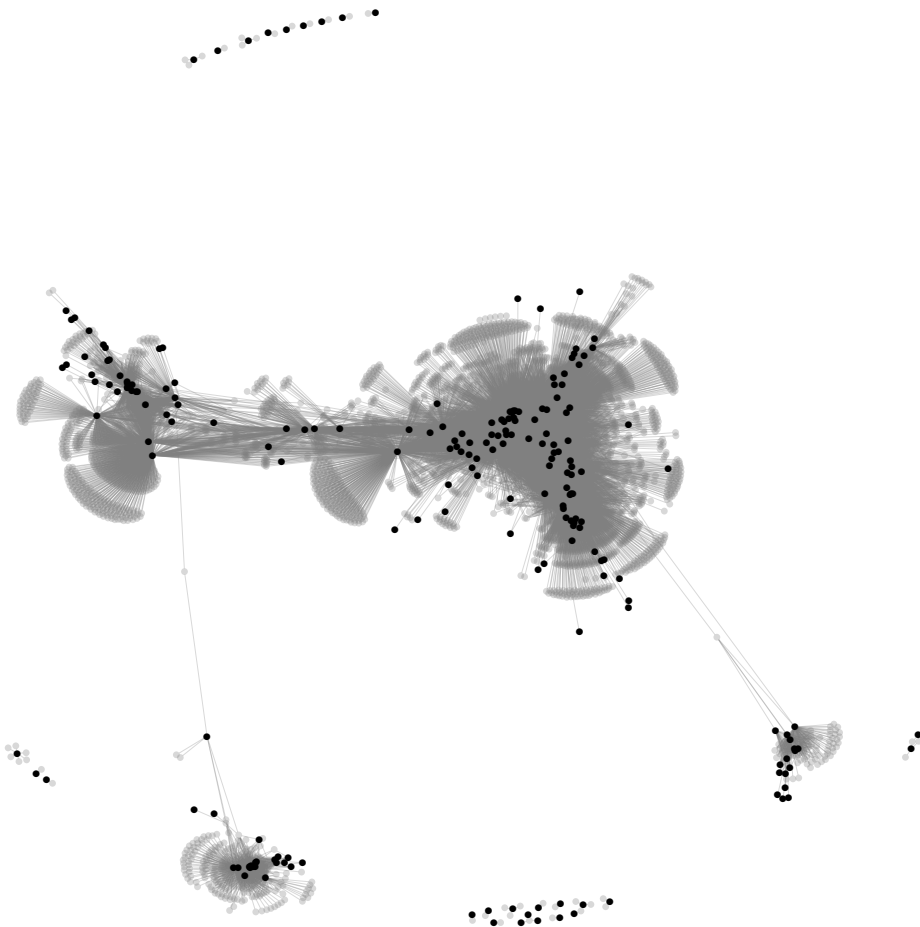

Supplement: Additional file 1: Figure S1. — Graph representation of the top correlation structure. Black and gray nodes represent gene expression and DNA methylation probes respectively. Edges represent correlations ≥0.5 that survived Bonferroni correction. The Pearson correlation between gene expression and DNA methylation was calculated based on levels unadjusted for cellular composition in 610 individuals from the BSGS dataset. The majority of the probes are connected to form the largest graph component. (PDF 2191 kb) [file 12864_2016_2498_MOESM1_ESM.pdf]

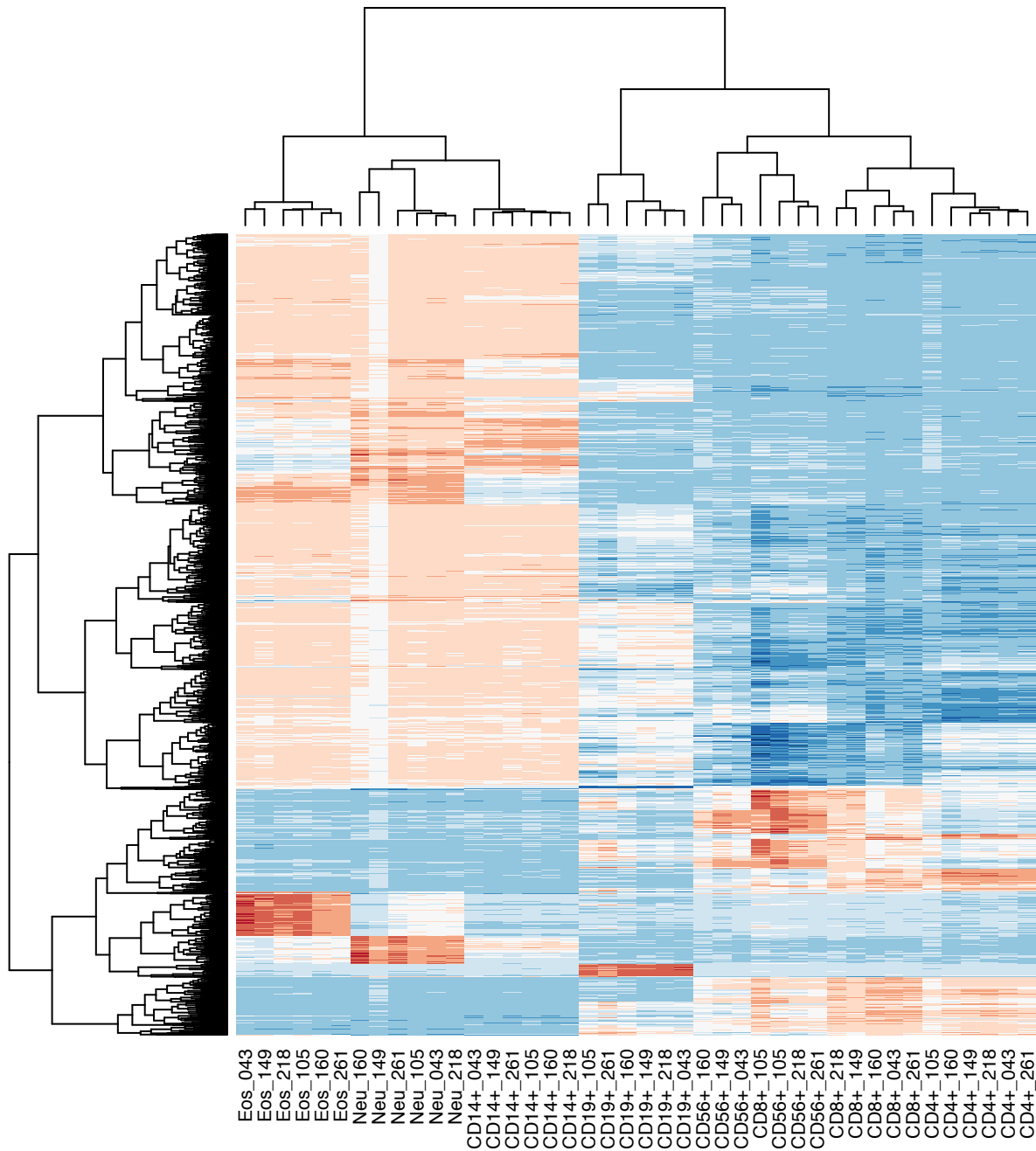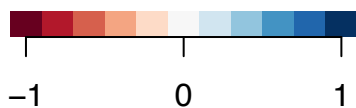

Supplement: Additional file 2: Figure S2. — Heatmap of DNA methylation (rows) matrix across purified hematopoietic cell types (columns). The methylation probes are selected based on the largest component of the top correlation graph (|ρ|≥0.5) before adjustment for the blood cellular composition. Hierarchical clustering separates samples into clusters according to their cell identity. The methylation data from Reinius et al. [19]. (PDF 1219 kb) [file 12864_2016_2498_MOESM2_ESM.pdf]

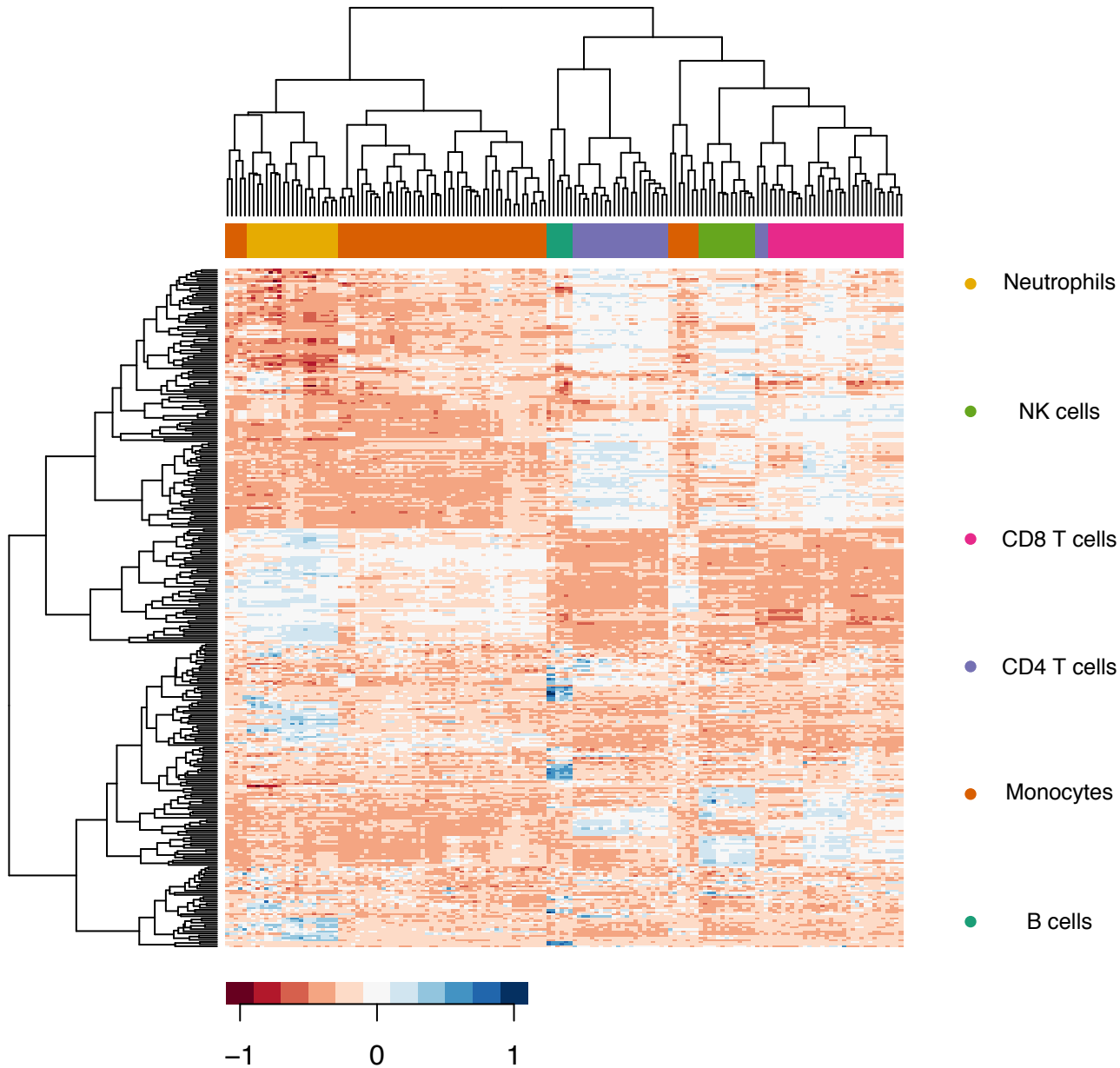

Supplement: Additional file 3: Figure S3. — Heatmap of gene expression (rows) matrix across purified hematopoietic cell types (columns). The genes are selected based on the largest component of the top correlation graph (|ρ|≥0.5) before adjustment for blood cellular composition. Hierarchical clustering separates samples into clusters according to their cell identity. Cell type identity of samples encoded by color bar at the top of the heatmap. The data from primary cell atlas [20]. (PDF 583 kb) [file 12864_2016_2498_MOESM3_ESM.pdf]

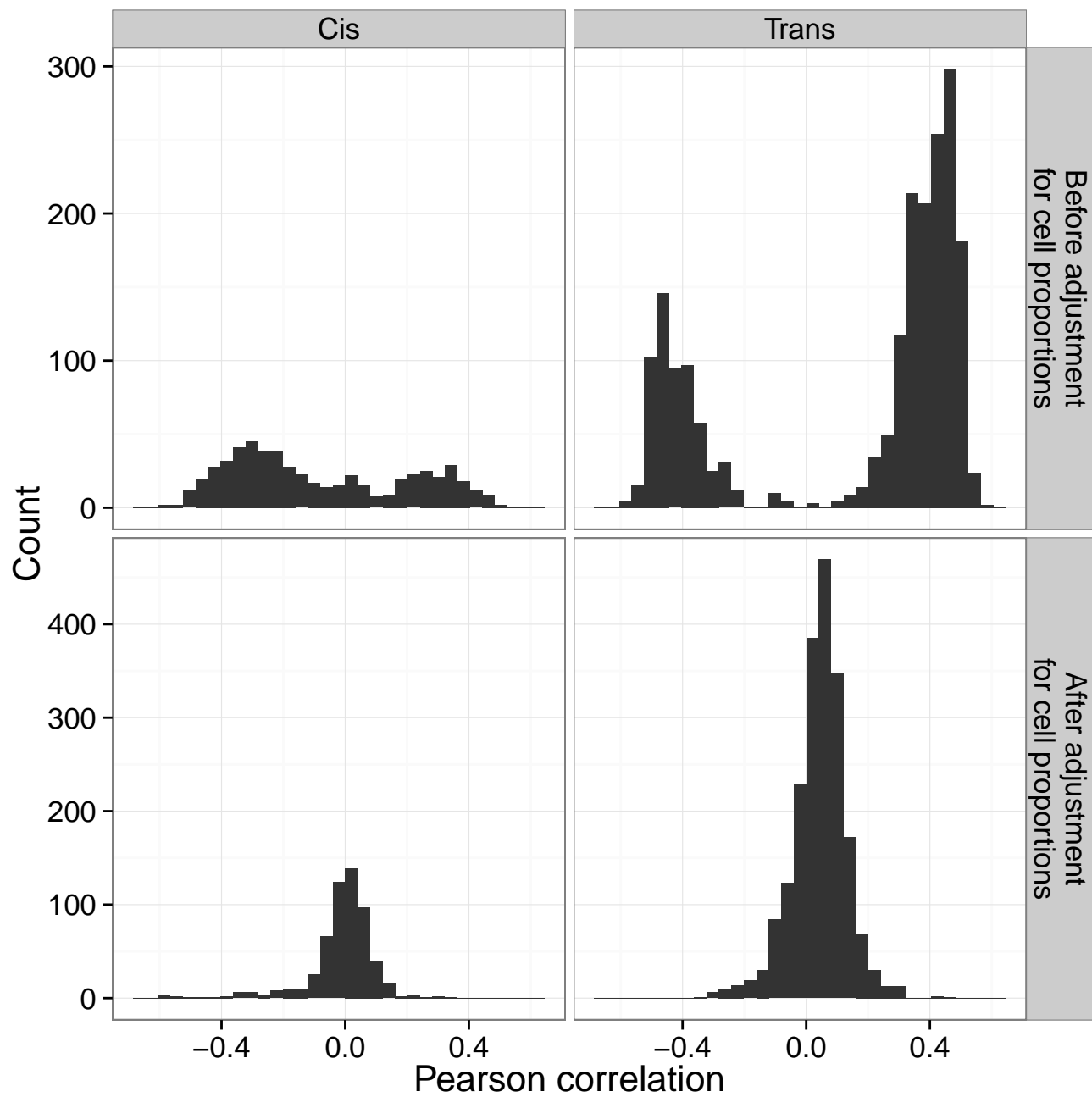

Supplement: Additional file 4: Figure S4. — Correlation look up of previously published expression-methylation probe pairs. Pearson correlations between gene expression and DNA methylation before (610 individuals) and after (422 individuals) adjustment for cellular composition in the BSGS dataset for cis and trans probe pairs that showed significant association in Eijk et al. [11]. The majority of the correlations shifted toward zero upon the adjustment for cellular composition. (PDF 6 kb) [file 12864_2016_2498_MOESM4_ESM.pdf]

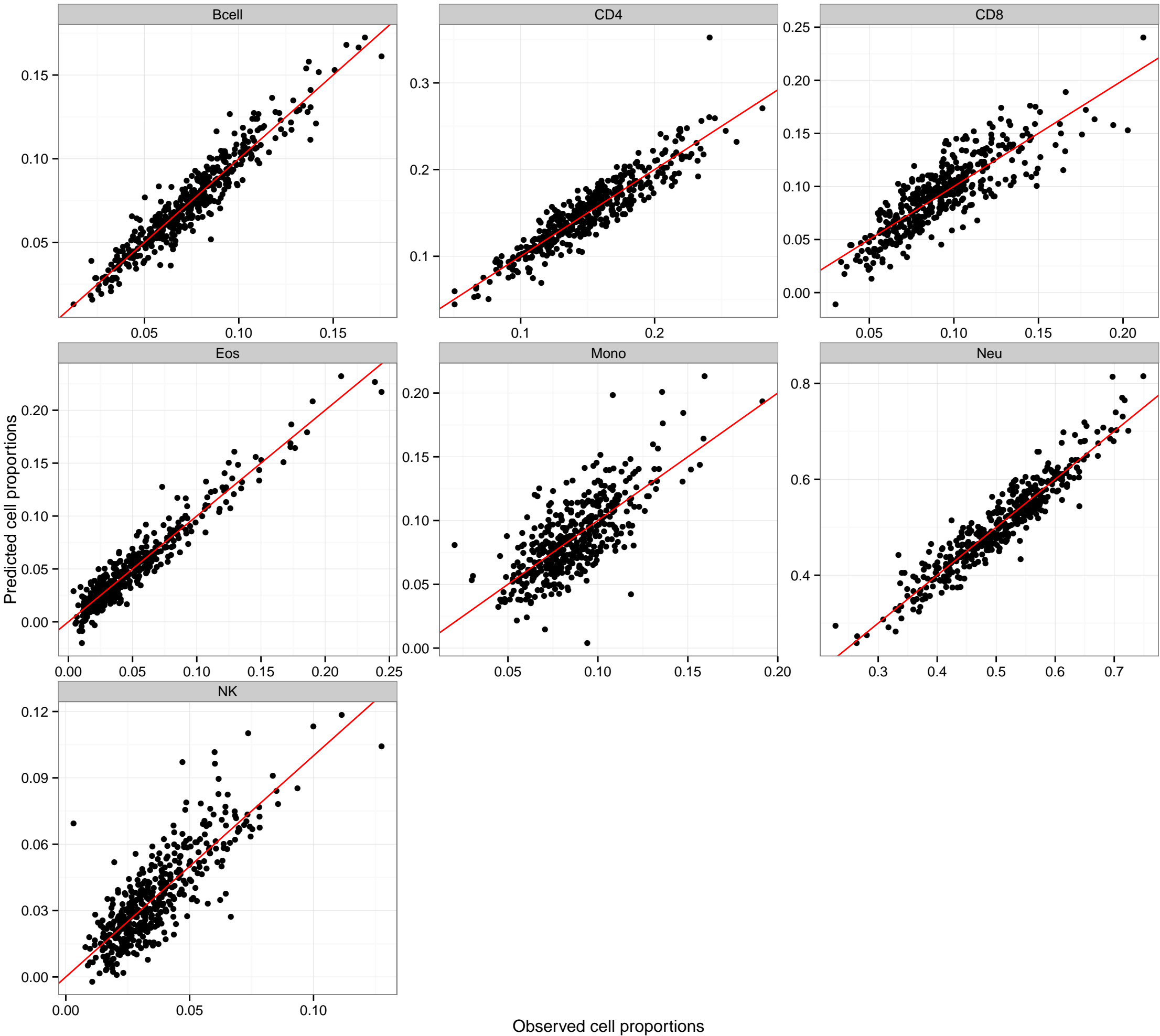

Supplement: Additional file 5: Figure S5. — Relationship between the observed (x-axis) and predicted (y-axis) cell proportions in the BSGS dataset split by cell type. Cell proportions were predicted utilizing methylation data with Houseman et al. [21] method re-trained on Reinius et al. [19] dataset. The predicted proportions were calibrated on the 422 subsample that have cellular composition measured to have a slope equal one and an intercept equal zero when regressed on observed proportions (see Table S1 for correlations between observed and predicted proportions). The red line is an identity line. (PDF 27 kb) [file 12864_2016_2498_MOESM5_ESM.pdf]

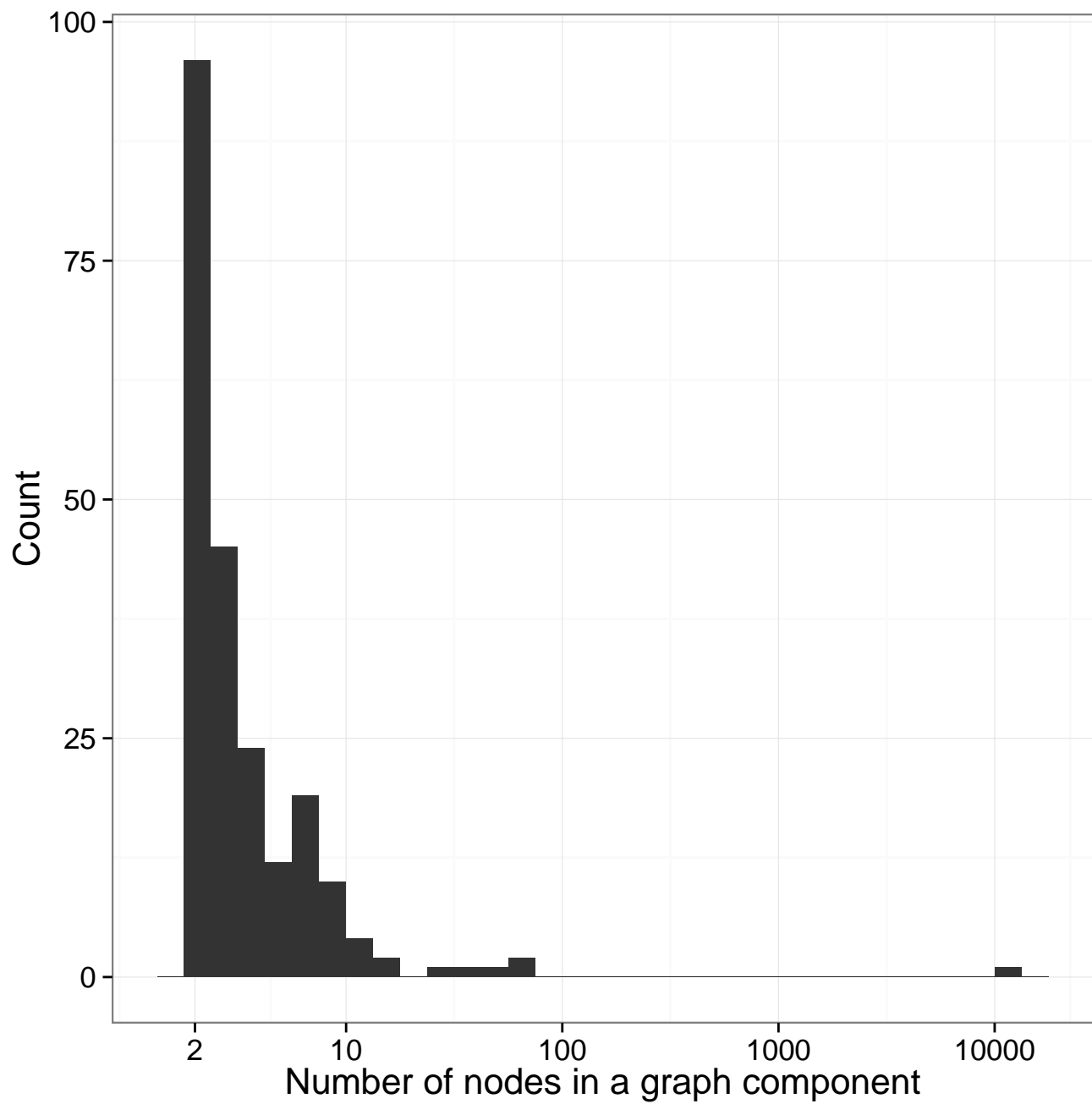

Supplement: Additional file 7: Figure S6. — Distribution of the number of nodes per graph component. Expression and methylation levels were adjusted for predicted cell proportions. The correlation graph was constructed from probe pairs (60275 probe pairs) passing Bonferroni correction. The largest component consists of 11251 nodes. (PDF 4 kb) [file 12864_2016_2498_MOESM7_ESM.pdf]

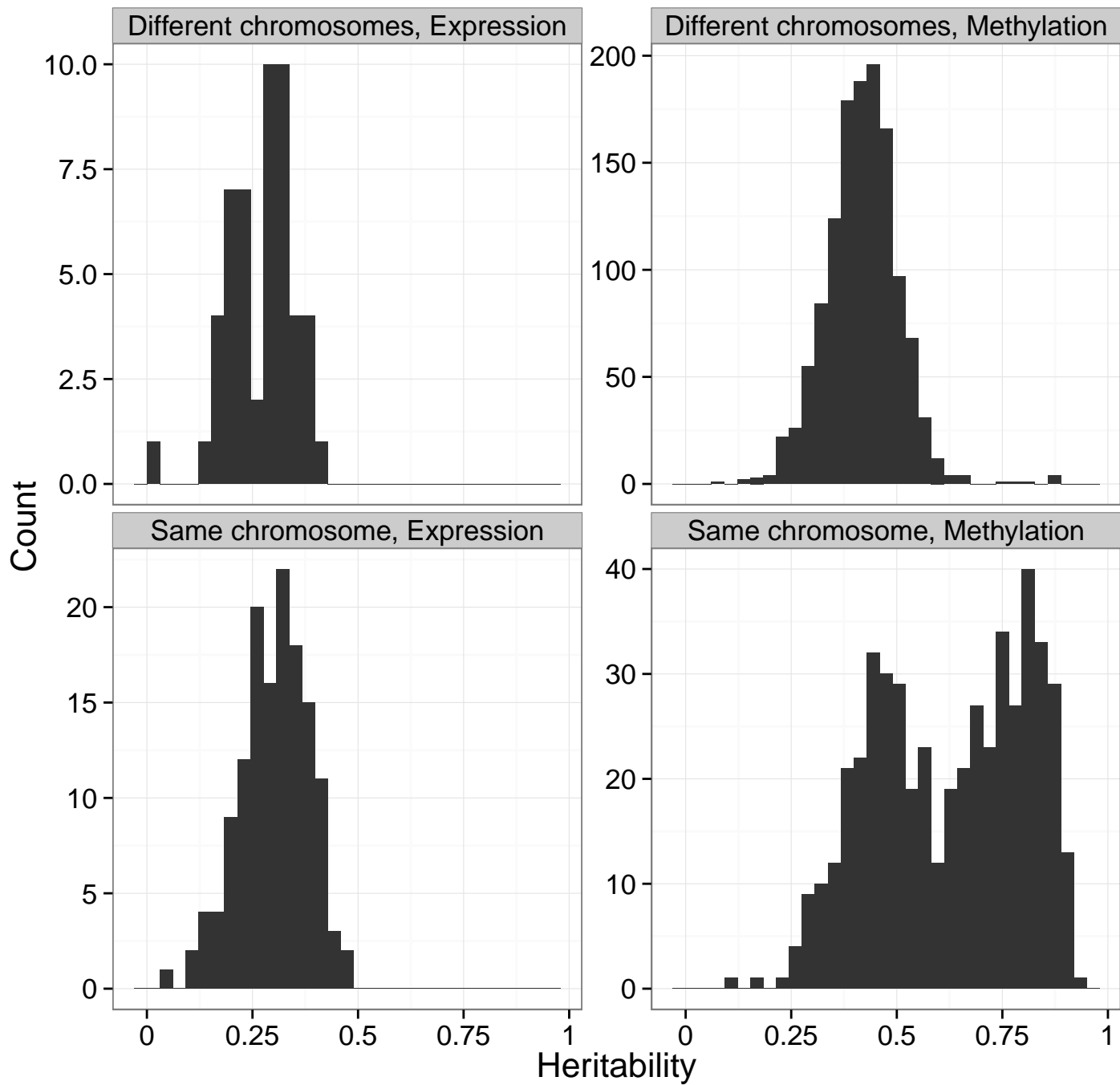

Supplement: Additional file 12: Figure S7. — Distribution of the heritability of expression and methylation probes from the final list of correlated probe pairs (3321 probe pairs). Unique expression and methylation probes were extracted from 2707 different chromosome and 614 same chromosome probe pairs. (PDF 6 kb) [file 12864_2016_2498_MOESM12_ESM.pdf]

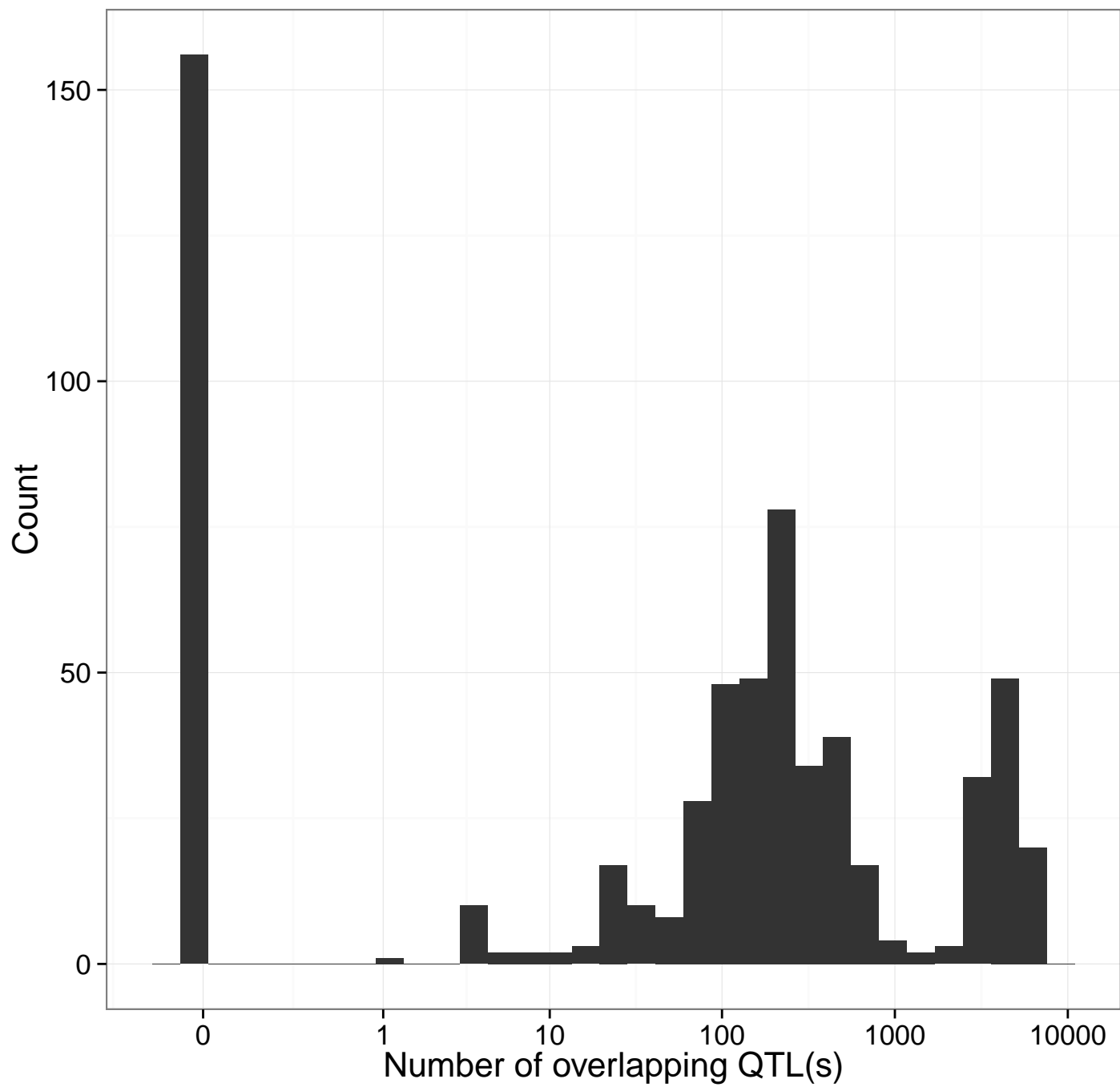

Supplement: Additional file 13: Figure S8. — Distribution of number of overlapping QTLs per probe pair. For each of the 614 same chromosome probe pairs, expression and methylation QTLs at nominal 10-5 p-value threshold were selected. The number of overlapping SNP was counted for each probe pair. 156 pairs do not share QTL. (PDF 4 kb) [file 12864_2016_2498_MOESM13_ESM.pdf]

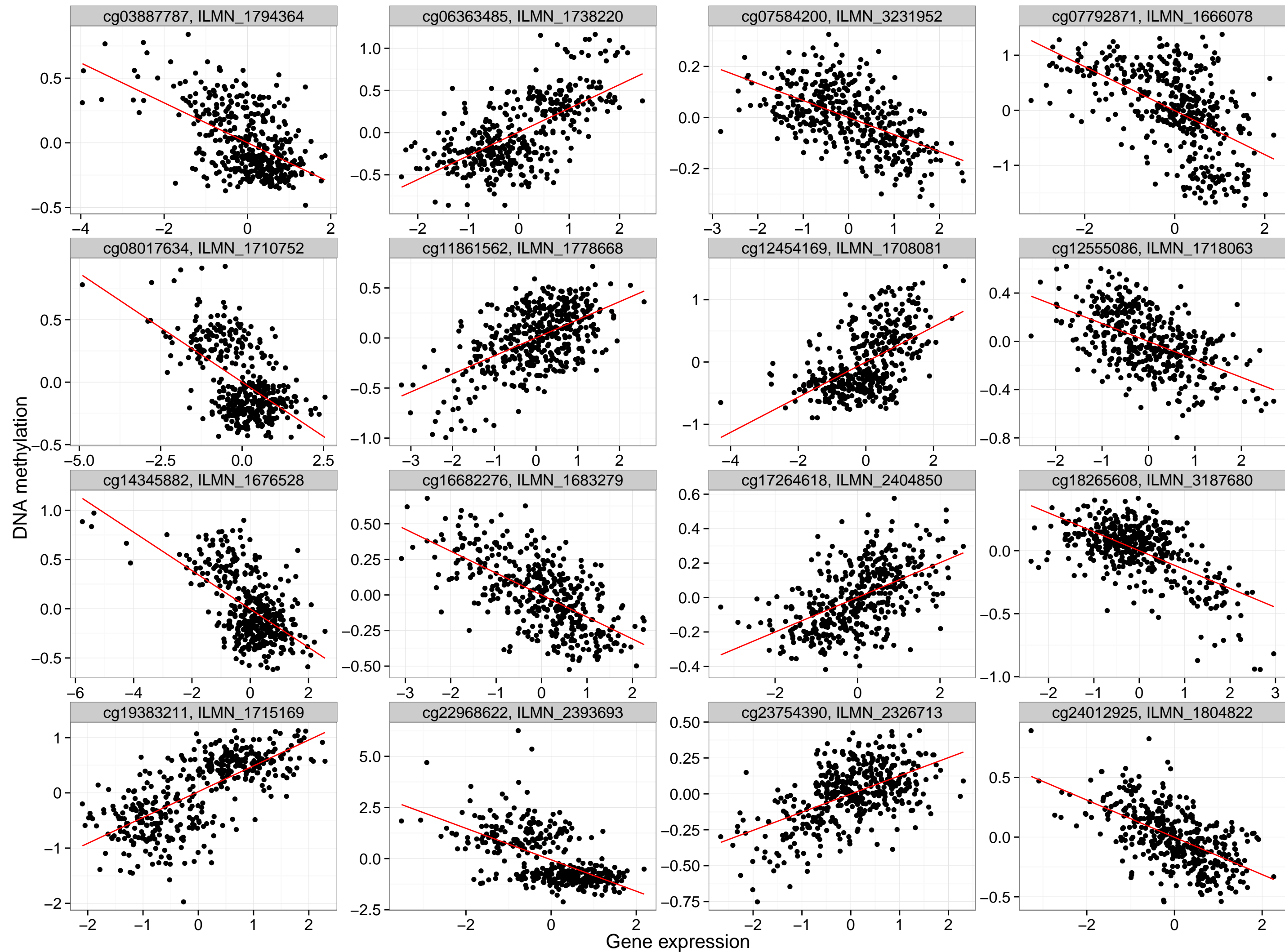

Supplement: Additional file 14: Figure S9. — Scatterplot of gene expression and DNA methylation for top sixteen same chromosome probe pairs with shared QTL(s). Red line is a linear regression of DNA methylation on gene expression values. (PDF 65 kb) [file 12864_2016_2498_MOESM14_ESM.pdf]

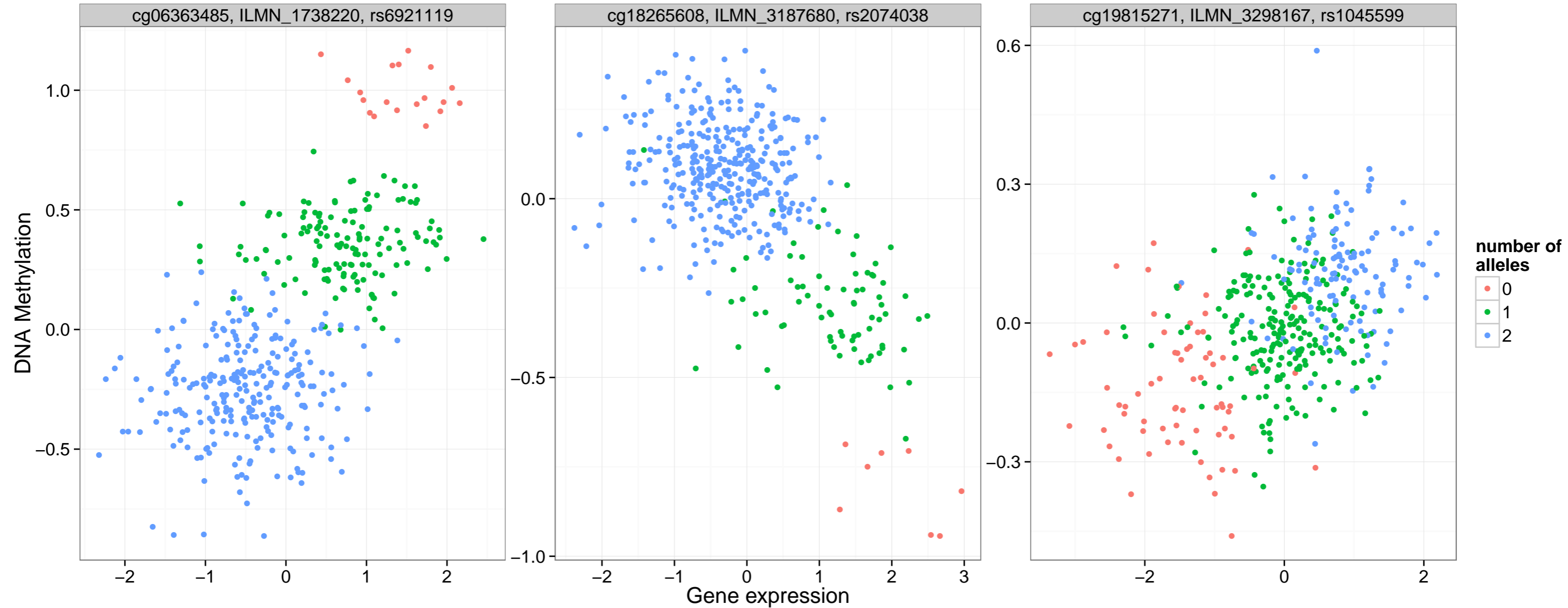

Supplement: Additional file 15: Figure S10. — Scatterplot of gene expression and DNA methylation for same chromosome probe pairs with shared QTL(s). Color represents number of alleles of e/mSNP shared by a DNA methylation gene expression probe pair. (PDF 16 kb) [file 12864_2016_2498_MOESM15_ESM.pdf]

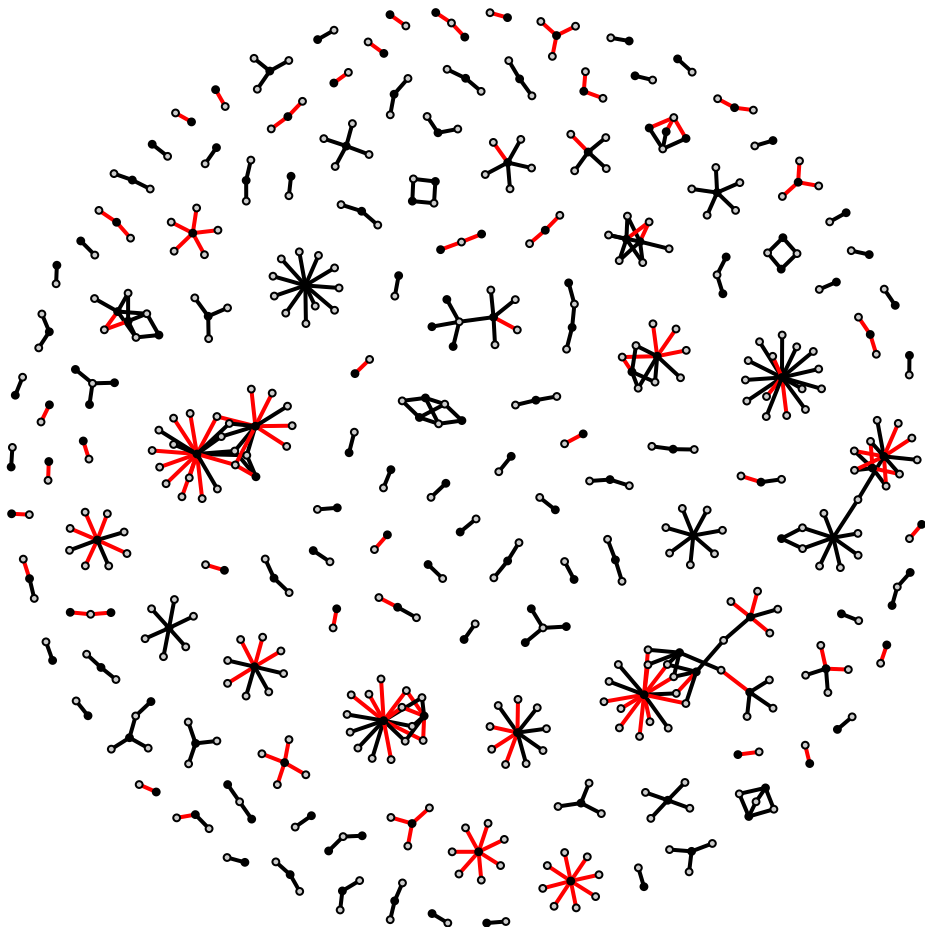

Supplement: Additional file 17: Figure S11. — Graph representation of the correlation structure for the same chromosome probe pairs with shared QTL(s). The graph is constructed by utilizing selected probes from the final correlation list. There are 135 components each of which correspond to a unique genomic location tagged by probes. 125 components correspond to a single gene. Expression and methylation probes are represented by black and grey nodes respectively. Positive and negative correlations are depicted as red and black edges respectively. (PDF 38 kb) [file 12864_2016_2498_MOESM17_ESM.pdf]

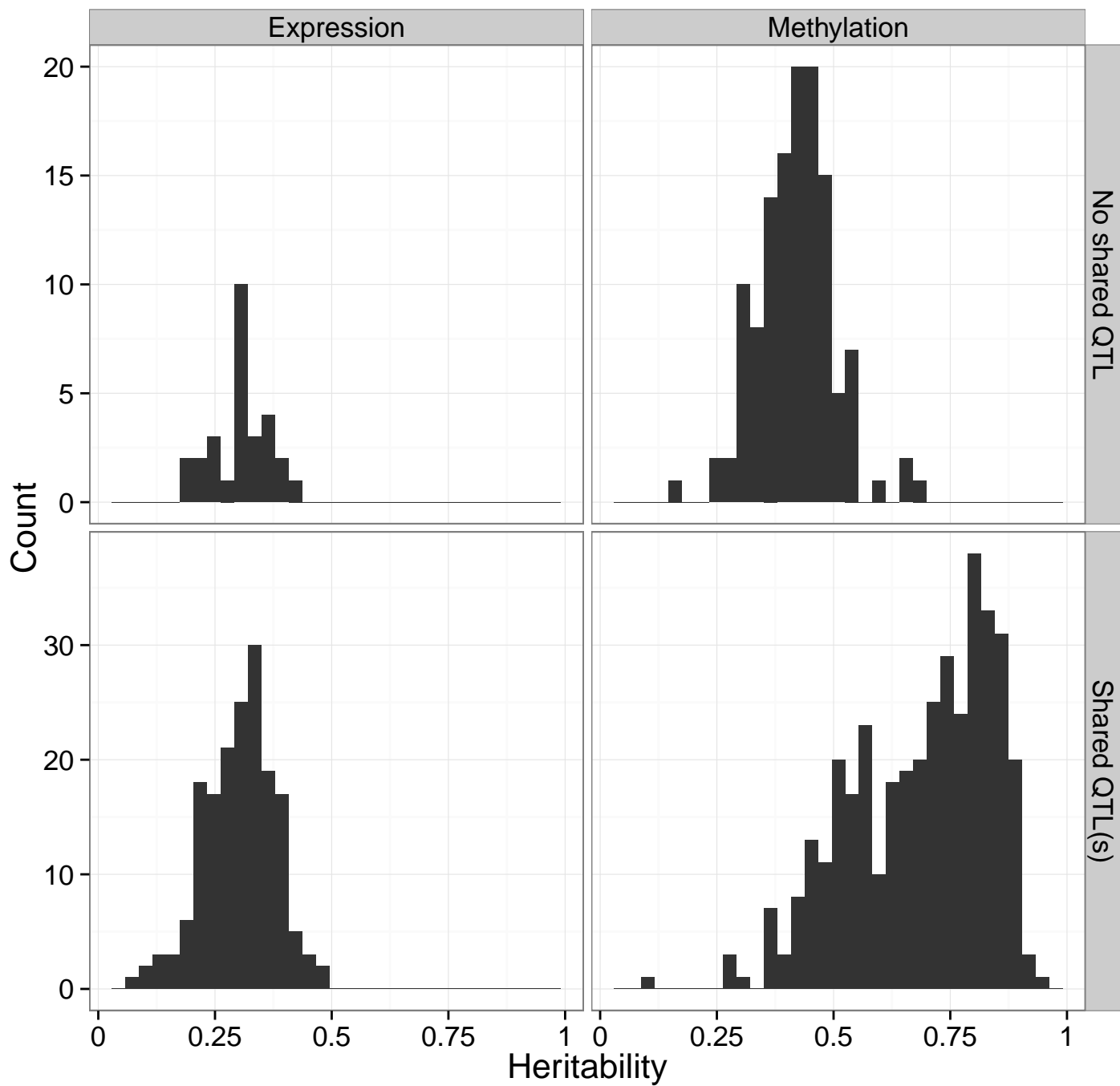

Supplement: Additional file 18: Figure S12. — Distribution of heritability of expression and methylation probes from same chromosome probe pairs (614 probe pairs, the final correlation list) split by shared QTL(s) status. Unique methylation and expression probes were extracted from 458 same chromosome shared QTL(s) and 156 same chromosome no shared QTL probe pairs. (PDF 5 kb) [file 12864_2016_2498_MOESM18_ESM.pdf]

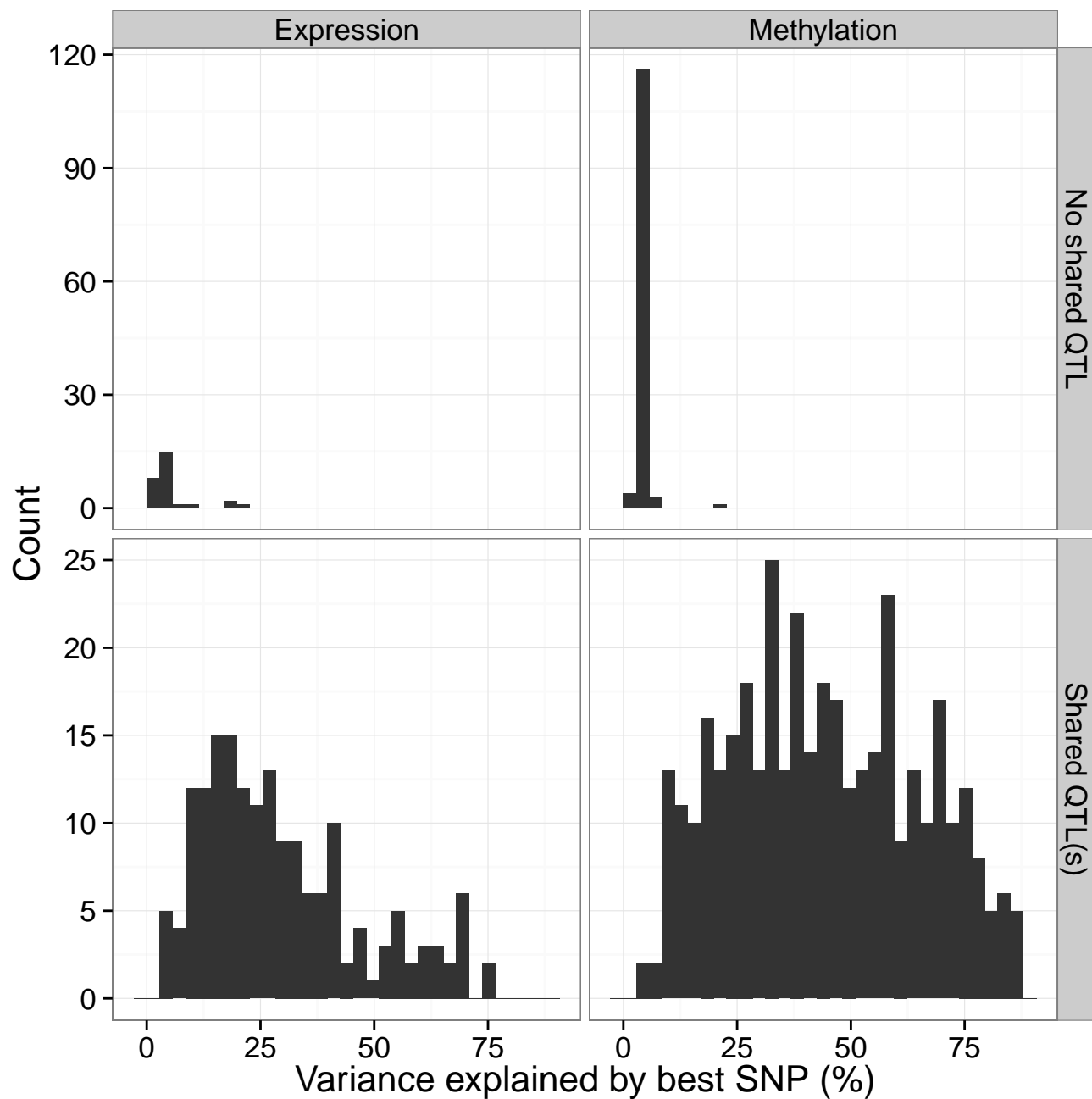

Supplement: Additional file 21: Figure S13. — Distribution of probe variance explained by the best SNP. For each unique expression and methylation probe from the same chromosome probe pair list (614 probe pairs) best expression and methylation association SNP respectively were selected. Probes were split based on the shared QTL status of the probe pair they originate from. (PDF 6 kb) [file 12864_2016_2498_MOESM21_ESM.pdf]

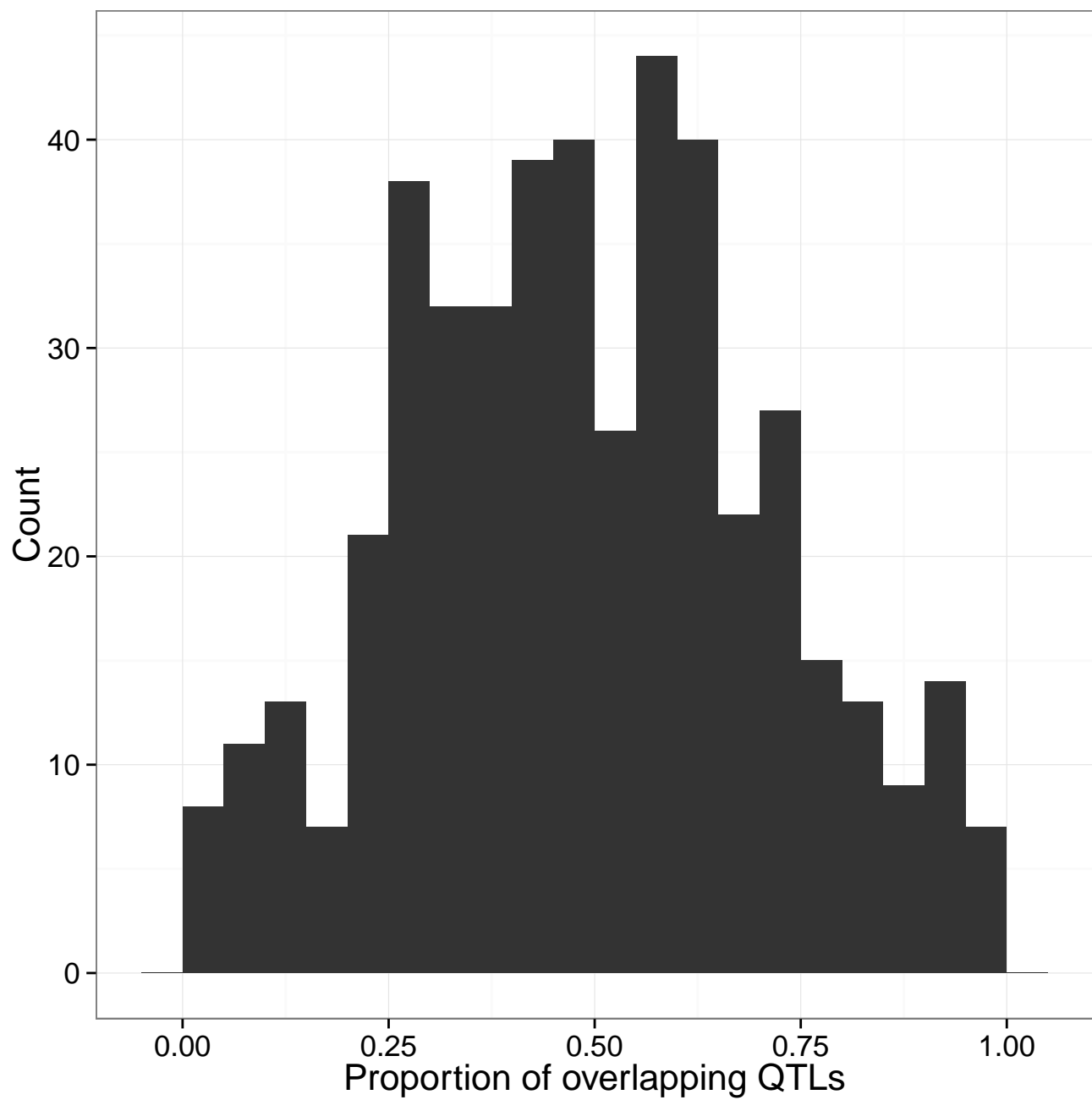

Supplement: Additional file 22: Figure S14. — Distribution of proportion of shared QTLs per probe pair. The 458 same chromosome probe pairs with shared QTL(s) from the final correlation list were selected. The proportion was calculated as the ratio of number expression and methylation association SNPs with the same rs id number at 10-5 p-value cutoff (shared QTL(s)) to the number of all unique m and e SNPs at the same threshold per probe pair. (PDF 4 kb) [file 12864_2016_2498_MOESM22_ESM.pdf]

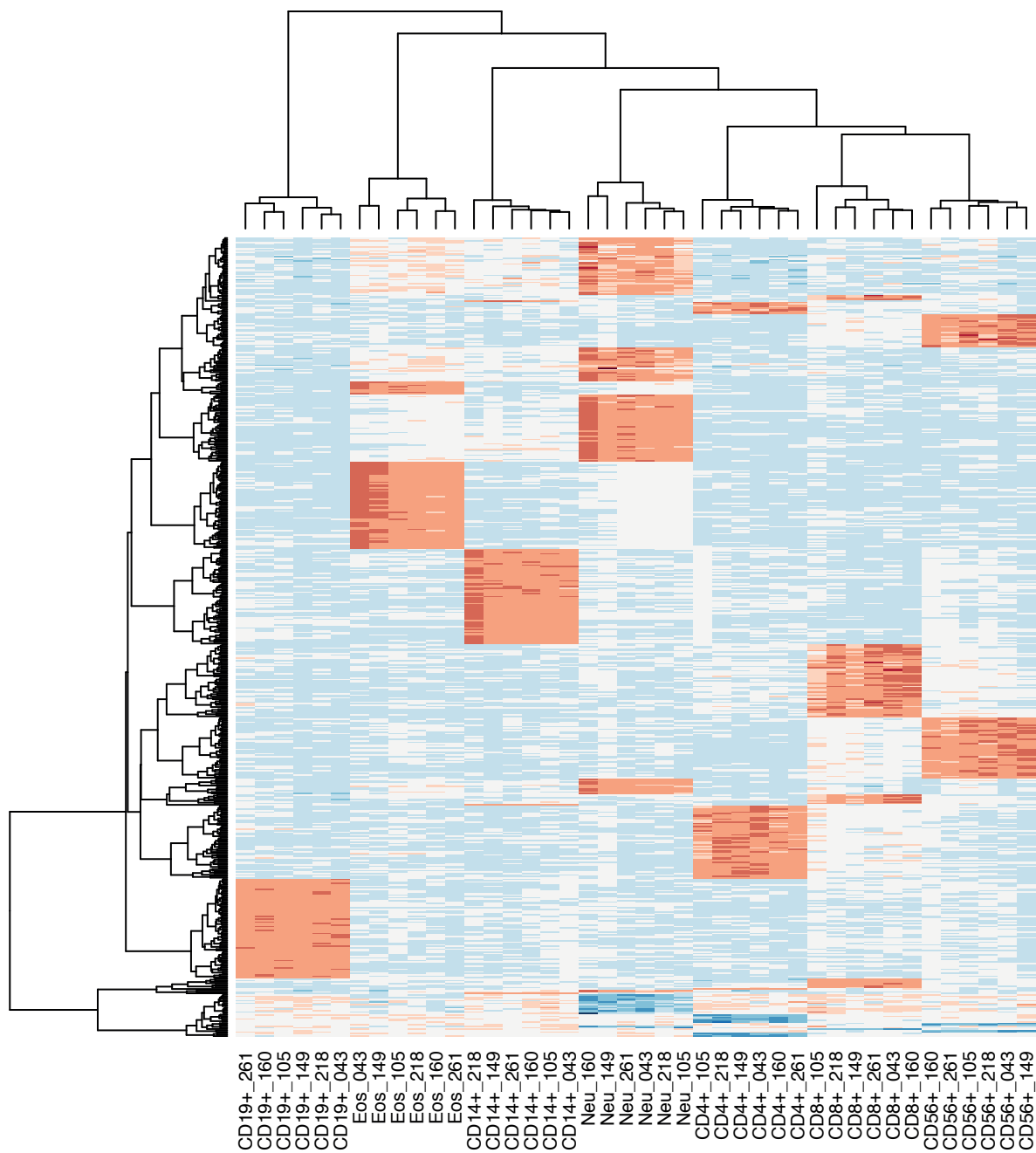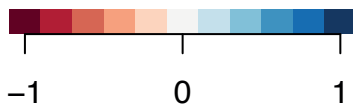

Supplement: Additional file 24: Figure S15. — Heatmap of DNA methylation (rows) matrix across purified hematopoietic cell types (columns). The methylation probes are selected based on differential methylation calls between a given cell type and the rest of the cell types. Probes with rank smaller or equal 70 selected. The methylation data from Reinius et al. [19]. (PDF 895 kb) [file 12864_2016_2498_MOESM24_ESM.pdf]
