# Supplementary material for: Shared genetic control of expression and methylation in peripheral blood
Source: BMC Genomics. 2016 Apr 6;17:278. doi: 10.1186/s12864-016-2498-4 (PMC4822256; doi:10.1186/s12864-016-2498-4)
Supplement: Additional file 6: Table S1. — Pearson correlations between observed and predicted cell proportions in the 422 individuals subset of the BSGS dataset. The predicted cell proportions are estimated via Houseman et al. method [21] which was retrained on Reinius et al. data [19]. (DOC 27 kb) [file 12864_2016_2498_MOESM6_ESM.doc]

| Cell Type | Correlation |
| --- | --- |
| CD19 B cell | 0.95 |
| CD4 T cells | 0.92 |
| CD8 T cells | 0.84 |
| Eosinophils | 0.96 |
| Monocytes | 0.72 |
| NK cells | 0.83 |
| Neutrophils | 0.94 |

Table S1: Pearson correl**a**tions between observed and predicted cell proportions in the 422 individuals subset of the BSGS dataset. The predicted cell proportions are estimated via Houseman et al. method[21] which was retrained on Reinius et al. data[19].
