# Supplementary material for: Shared genetic control of expression and methylation in peripheral blood
Source: BMC Genomics. 2016 Apr 6;17:278. doi: 10.1186/s12864-016-2498-4 (PMC4822256; doi:10.1186/s12864-016-2498-4)
Supplement: Additional file 11: Table S2. — Number of unique DNA methylation probes correlated with a gene in the largest correlation graph component. Probe pairs from the largest graph component of the final correlation list were selected (see Fig. 4). Unique methylation probes per gene were counted such that a methylation probe was counted only once if it correlated with more than one expression probe tagging the same gene. (DOC 33 kb) [file 12864_2016_2498_MOESM11_ESM.doc]

| Gene Name | Number of methylation probes |
| --- | --- |
| GZMH | 1085 |
| CCL5 | 645 |
| GPR56 | 229 |
| KLRG1 | 102 |
| KLRD1 | 53 |
| LAG3 | 52 |
| NKG7 | 34 |
| S1PR5 | 33 |
| TGFBR3 | 25 |
| MIAT | 18 |
| PPP2R2B | 18 |
| MCOLN2 | 17 |
| FGFBP2 | 11 |
| FLJ33590 | 6 |
| GNLY | 4 |
| TARP | 4 |
| KLRB1 | 3 |
| APOBEC3G | 2 |
| CST7 | 2 |
| FLJ20699 | 2 |
| PRF1 | 2 |
| ASCL2 | 1 |
| AUTS2 | 1 |
| LYAR | 1 |
| PATL2 | 1 |
| RORC | 1 |
| TTC38 | 1 |

Table S2: Number of unique DNA methylation probes correlated with a gene in the largest correlation graph component. Probe pairs from the largest graph component of the final correlation list were selected (see Figure 4). Unique methylation probes per gene were counted such that a methylation probe was counted only once if it correlated with more than one expression probe tagging the same gene.
