# Supplementary material for: Shared genetic control of expression and methylation in peripheral blood
Source: BMC Genomics. 2016 Apr 6;17:278. doi: 10.1186/s12864-016-2498-4 (PMC4822256; doi:10.1186/s12864-016-2498-4)
Supplement: Additional file 23: Table S3. — The number of the same chromosome probes pairs with shared QTL(s) from the final correlation list that contribute towards different graph components stratified by the adjustment methods. The last row of the table is the final correlation graph (Fig. 4). (DOC 27 kb) [file 12864_2016_2498_MOESM23_ESM.doc]

| Adjustment method | Largest component | Rest of graph | Total |
| --- | --- | --- | --- |
| No correction | 301 | 113 | 414 |
| Predicted proportions | 7 | 444 | 451 |
| Observed proportions | 11 | 447 | 458 |

Table S3: The number of the same chromosome probes pairs with shared QTL(s) from the final correlation list that contribute towards different graph components stratified by the adjustment methods. The last row of the table is the final correlation graph (Figure 4).
