# Supplementary material for: Shared genetic control of expression and methylation in peripheral blood
Source: BMC Genomics. 2016 Apr 6;17:278. doi: 10.1186/s12864-016-2498-4 (PMC4822256; doi:10.1186/s12864-016-2498-4)
Supplement: Additional file 25: — Modeling of correlation induced by differential cell counts and column description of the Tables S4-S9. (DOCX 90 kb) [file 12864_2016_2498_MOESM25_ESM.pdf]

Shared genetic control of expression and methylation in peripheral blood.

Konstantin Shakhbazov[1,+], Joseph E. Powell[1,2,3], Gibran Hemani[1,**&**], Anjali K. Henders[1], Nicholas G. Martin[3], Peter M. Visscher[1,4,*], Grant W. Montgomery[3,*], Allan F. McRae[1,4,*]

1. Queensland Brain Institute, University of Queensland, Brisbane, QLD, Australia

2. The Institute for Molecular Bioscience, University of Queensland, Brisbane, QLD, Australia

3. QIMR Berghofer Medical Research Institute, Royal Brisbane Hospital, QLD, Australia

4. University of Queensland Diamantina Institute, Translational Research Institute, Brisbane, QLD, Australia

& Current address: MRC Integrative Epidemiology Unit and School of Social and Community Medicine, University of Bristol, Bristol, UK, BS8 2BN

***** These authors contributed equally

+ Corresponding author: k.shakhbazov@uq.edu.au

Supplemental Methods

Modeling of correlation induced by differential cell counts

To get better understanding of the correlation between gene expression and DNA methylation induced by differential cell counts the methylation, expression and cell proportion for a given probe pair and a cell type were modeled as a trivariate normal (of course cell proportions are not normal, beta distribution would be more appropriate but normal is a good approximation). Let E, M and C represent random variables for expression, methylation and cell proportion. Assume no correlation between expression and methylation within a particular cell type, that is to say observed correlation between E and M is solely due to differential cell counts. Zero correlation between E and M corrected for C equivalent to zero correlation between E and M given C. Then correlation between E and M given C utilizing predictor for C $\hat{C}$ with a correlation between C and $\hat{C}$: $r_{C\hat{C}}$ is

$$r_{\hat{E}}=r_{EC}r_{MC}(1-r_{C\hat{C}}^{2}).$$

Where $r_{EC}r_{MC}$ is the correlation between expression and methylation not corrected for cellular composition (follows from $cor(E,M|C)=0$). Given the range of $r_{C\hat{C}}$ which we observed from comparison of observed and predicted proportions in the 422 sub-sample ≈0.75−0.95 (Table S1), after correction with predicted cell proportion we left with 10 to 44 % of original unadjusted correlation between expression and methylation ($r_{EC}r_{MC}$) unlike in case of adjustment with observed cell proportion where $cor(E,M|C)=0$.

Table S4: The 3321 probe pairs from the final correlation list. Phenotypic Person correlation and genetic correlations between expression and methylation probes, related statistics and heritabilities are provided.

File: table_s4.csv

- meth_ind: Illumina 450K Methylation probe ID
- exp_ind: Illumina HT12v4 Expression probe ID
- pearson: Pearson correlation coefficient
- pval: pvalue of Pearson correlation coefficient not being equal zero
- sam_size: sample size after removing all pairwise missing values
- h2_expression: heritability of an expression probe
- h2_methylation: heritability of a methylation probe
- rg: genetic correlation
- rg_se: standard error of the genetic correlation
- same_chromosome: indicates if expression and methylation probes located on the same chromosome
- qtls_status: indicates if expression and methylation probes share any association SNPs at 10^{-5} threshold
- dist: distance in bp between expression and methylation probes on the hg19 genome assembly

Table S5: Annotation and genomic position of the expression probes from the final correlation list.

File: table_s5.csv

- exp_ind: Illumina HT12v4 Expression probe ID
- gene_name: HGNC name of the gene tagged by the expression probe
- gencode18_id: Genocode version 18 id of the gene tagged by the expression probe
- gene_type: type of the gene (e.g. protein coding, lincRNA etc)
- chr: chromosome on which probe is located
- start: bp position on the hg19 genome assembly demarcating start of the expression probe (spliced probes correspond to multiple rows)
- end: bp position on the hg19 genome assembly demarcating end of the expression probe (spliced probes correspond to multiple rows)
- width: length in bp of the genome region between start and end of the probe (50bp, should sum up to 50 in case of spliced probes)
- strand: direction of the probe (i.e. this should be used to infer direction of the probe, NOT start or end positions above)

Table S6: Genomic position of the methylation probes from the final correlation list.

File: table_s6.csv

- meth_ind: Illumina 450K Methylation probe ID
- chr: chromosome on which probe is located
- probeStart: bp position on the hg19 genome assembly demarcating start of the methylation probe
- probeEnd: bp position on the hg19 genome assembly demarcating end of the methylation probe
- probeTarget: bp position on the hg19 genome assembly at which DNA methylation is measured

Table S7: Results of the association analysis between shared e/mSNPs and same chromosome probe pairs with a shared QTL(s). After selecting all the overlapping SNPs at nominal 10^{-5} association p-value threshold, we pruned the list to contain only SNPs with the lowest p-value from both the expression and methylation association analysis.

File: table_s7.csv

- meth_ind: Illumina 450K Methylation probe ID
- exp_ind: Illumina HT12v4 Expression probe ID
- SNP: rs id number
- CHR_meth: SNP chromosome location on the hg19 genome assembly
- AL1_meth: First allele (association with a methylation probe)
- AL2_meth: Second allele (association with a methylation probe)
- FREQ1_meth: frequency of the first allele (association with a methylation probe)
- EFFECT_meth: effect size of a SNP (association with a methylation probe)
- SE_meth: standard error of the effect size (association with a methylation probe)
- H2_meth: variance explained by a SNP (association with a methylation probe)
- LOD_meth: log odds ratio (association with a methylation probe)
- PVALUE_meth: p-value of the association between a SNP and a methylation probe not being equal zero.
- CHR_expr: SNP chromosome location on the hg19 genome assembly
- AL1_expr: First allele (association with an expression probe)
- AL2_expr: Second allele (association with an expression probe)

- FREQ1_expr: frequency of the first allele (association with an expression probe)
- EFFECT_expr: effect size of a SNP (association with an expression probe)
- SE_expr: standard error of the effect size (association with an expression probe)
- H2_expr: variance explained by a SNP (association with an expression probe)
- LOD_expr: log odds ratio (association with an expression probe)
- PVALUE_expr: p-value of the association between a SNP and an expression probe not being equal zero.

Table S8: Best association eSNPs for the expression probes from the final correlation list.

File: table_s8.csv

- exp_ind: Illumina HT12v4 Expression probe ID
- SNP: rs id number
- CHR_expr: SNP chromosome location on the hg19 genome assembly
- AL1_expr: First allele
- AL2_expr: Second allele
- FREQ1_expr: frequency of the first allele
- EFFECT_expr: effect size of a SNP
- SE_expr: standard error of the effect size
- H2_expr: variance explained by a SNP
- LOD_expr: log odds ratio
- PVALUE_expr: p-value of the association between a SNP and a probe not being equal zero.

Table S9: Best association mSNPs for the methylation probes from the final correlation list.

File: table_s9.csv

- meth_ind: Illumina 450K Methylation probe ID
- SNP: rs id number
- CHR_meth: SNP chromosome location on the hg19 genome assembly
- AL1_meth: First allele
- AL2_meth: Second allele
- FREQ1_meth: frequency of the first allele
- EFFECT_meth: effect size of a SNP

- SE_meth: standard error of the effect size
- H2_meth: variance explained by a SNP
- LOD_meth: log odds ratio
- PVALUE_meth: p-value of the association between a SNP and a probe not being equal zero.
